# Antigen pressure from two founder viruses induces multiple insertions at a single antibody position to generate broadly neutralizing HIV antibodies

**DOI:** 10.1371/journal.ppat.1011416

**Published:** 2023-06-29

**Authors:** Collin Joyce, Sasha Murrell, Ben Murrell, Oluwarotimi Omorodion, Lorena S. Ver, Nancy Carrico, Raiza Bastidas, Rebecca Nedellec, Michael Bick, Jordan Woehl, Fangzhu Zhao, Alison Burns, Shawn Barman, Michael Appel, Alejandra Ramos, Lalinda Wickramasinghe, Kemal Eren, Thomas Vollbrecht, Davey M. Smith, Sergei L. Kosakovsky Pond, Ryan McBride, Charli Worth, Facundo Batista, Devin Sok, The IAVI Protocol C Investigators & The IAVI African HIV Research Network, Pascal Poignard, Bryan Briney, Ian A. Wilson, Elise Landais, Dennis R. Burton

**Affiliations:** 1 Department of Immunology and Microbiology, The Scripps Research Institute, La Jolla, California, United States of America; 2 Consortium for HIV/AIDS Vaccine Development (CHAVD), The Scripps Research Institute, La Jolla, California, United States of America; 3 IAVI Neutralizing Antibody Center, The Scripps Research Institute, La Jolla, California, United States of America; 4 Department of Integrative Structural and Computational Biology, The Scripps Research Institute, La Jolla, California, United States of America; 5 Department of Medicine, University of California San Diego, San Diego, California, United States of America; 6 Department of Microbiology, Tumor and Cell biology, Karolinska Institutet, Stockholm, Sweden; 7 IAVI, New York, New York, United States of America; 8 Veterans Affairs San Diego Healthcare System, San Diego, California, United States of America; 9 Institute for Genomics and Evolutionary Medicine, Temple University, Philadelphia, Pennsylvania, United States of America; 10 Department of Molecular Medicine, The Scripps Research Institute, La Jolla, California, United States of America; 11 Ragon Institute of MGH, MIT and Harvard, Cambridge, Massachusetts, United States of America; 12 Institut de Biologie Structurale, Université Grenoble Alpes, Commissariat à l’Energie Atomique, Centre National de Recherche Scientifique and Centre Hospitalier Universitaire Grenoble Alpes, Grenoble, France; 13 Center for Viral Systems Biology, The Scripps Research Institute, La Jolla, California, United States of America; 14 Skaggs Institute for Chemical Biology, The Scripps Research Institute, La Jolla, California, United States of America; Vaccine Research Center, UNITED STATES

## Abstract

Vaccination strategies aimed at maturing broadly neutralizing antibodies (bnAbs) from naïve precursors are hindered by unusual features that characterize these Abs, including insertions and deletions (indels). Longitudinal studies of natural HIV infection cases shed light on the complex processes underlying bnAb development and have suggested a role for superinfection as a potential enhancer of neutralization breadth. Here we describe the development of a potent bnAb lineage that was elicited by two founder viruses to inform vaccine design. The V3-glycan targeting bnAb lineage (PC39-1) was isolated from subtype C-infected IAVI Protocol C elite neutralizer, donor PC39, and is defined by the presence of multiple independent insertions in CDRH1 that range from 1-11 amino acids in length. Memory B cell members of this lineage are predominantly atypical in phenotype yet also span the class-switched and antibody-secreting cell compartments. Development of neutralization breadth occurred concomitantly with extensive recombination between founder viruses before each virus separated into two distinct population “arms” that evolved independently to escape the PC39-1 lineage. Ab crystal structures show an extended CDRH1 that can help stabilize the CDRH3. Overall, these findings suggest that early exposure of the humoral system to multiple related Env molecules could promote the induction of bnAbs by focusing Ab responses to conserved epitopes.

## Author summary

Broadly neutralizing antibodies (bnAbs) are capable of neutralizing multiple strains of a given virus. Reliable vaccine elicitation of bnAbs represents the most promising solution to stopping the HIV-1 pandemic. BnAbs bind to conserved regions of the HIV envelope glycoprotein (Env) and are typically isolated from infected individuals. Accordingly, an understanding of the complex processes underlying bnAb development may shed light on vaccine design. Here we studied the case of an individual living with HIV-1 who was infected with two similar related founder viruses and went on to mount a broadly neutralizing immune response after 9–11 months of infection. From this individual, we isolated a potent lineage of bnAbs that features different lengths of genetic insertions within a single position. By characterizing the antibody-virus co-evolution that occurs within this individual, we offer insight into how a bnAb response might be facilitated through vaccination.

## Introduction

Broadly neutralizing antibodies (bnAbs), i.e. those capable of neutralizing multiple strains of a given virus and typically isolated from infected individuals, have attracted much attention as prophylactic and therapeutic agents and as guides for vaccine design [1–9]. BnAbs are crucial in the design of vaccines for highly antigenically variable viruses such as HIV and influenza [6, 9–14]. For HIV, the development of bnAbs in infected individuals has been followed longitudinally for a number of donors [15–27]. It has become clear that bnAbs develop in natural infection as a result of the encounter of the humoral immune system with multiple different viruses corresponding to multiple different target Envelope (Env) molecules in a sequential fashion [13, 28, 29]. Typical Env molecules show little to no affinity for naïve precursor antibodies presented as receptors on the surface of B cells (BCRs) in the activation of bnAb lineages [12, 30–37]. Usually, an approximation of the naïve B cell-triggering antibody is determined by computational methods extrapolating back from later time points in infection. The naïve antibody is often referred to as the unmutated common ancestor (UCA) of the bnAb lineage. Antibodies isolated during the course of infection are then analyzed with the goal of finding clues as to a set of immunogens that, when used sequentially, would elicit bnAbs [13, 28, 29].

Longitudinal studies of bnAb development in HIV infection have highlighted the potential role of superinfection, which can be envisaged to enhance neutralization breadth by focusing antibody responses on epitopes conserved between the infecting and superinfecting viruses. Several studies have indeed suggested that superinfection broadens nAb responses [25, 38, 39], but other studies have not seen such an effect [40, 41]. One superinfected donor (CAP256) developed extremely potent bnAbs [18] but this was shown not to be a property of superinfection directing responses to epitopes conserved between infecting and superinfecting viruses but to a specific response to the superinfecting strain. Another superinfection study reported a similar result [42]. A further recent study associated the development of neutralization breadth with exposure to multiple closely related founder viruses [43]. Finally, one of the key elements in the development of many bnAbs is the incorporation of insertions and/or deletions (indels) into antibody sequences [44–46], which is typically viewed as a significant restriction on the induction of bnAbs through vaccination [13].

In this study, we investigated a donor who developed a family of potent HIV bnAbs with different lengths of an insertion in a critical framework region following infection with two founder viruses that were closely related and underwent extensive recombination. We used paired heavy-light chain isolation to generate antibodies at different phases of infection and coupled this with sequence studies on viral evolution and structural studies on the antibodies. We conclude that simultaneous exposure of the humoral system to multiple closely related Envs relatively early in infection may assist in the development of neutralization breadth.

## Results

### Isolation of a V3-glycan broadly neutralizing antibody (bnAb) lineage

**Fig 1:**
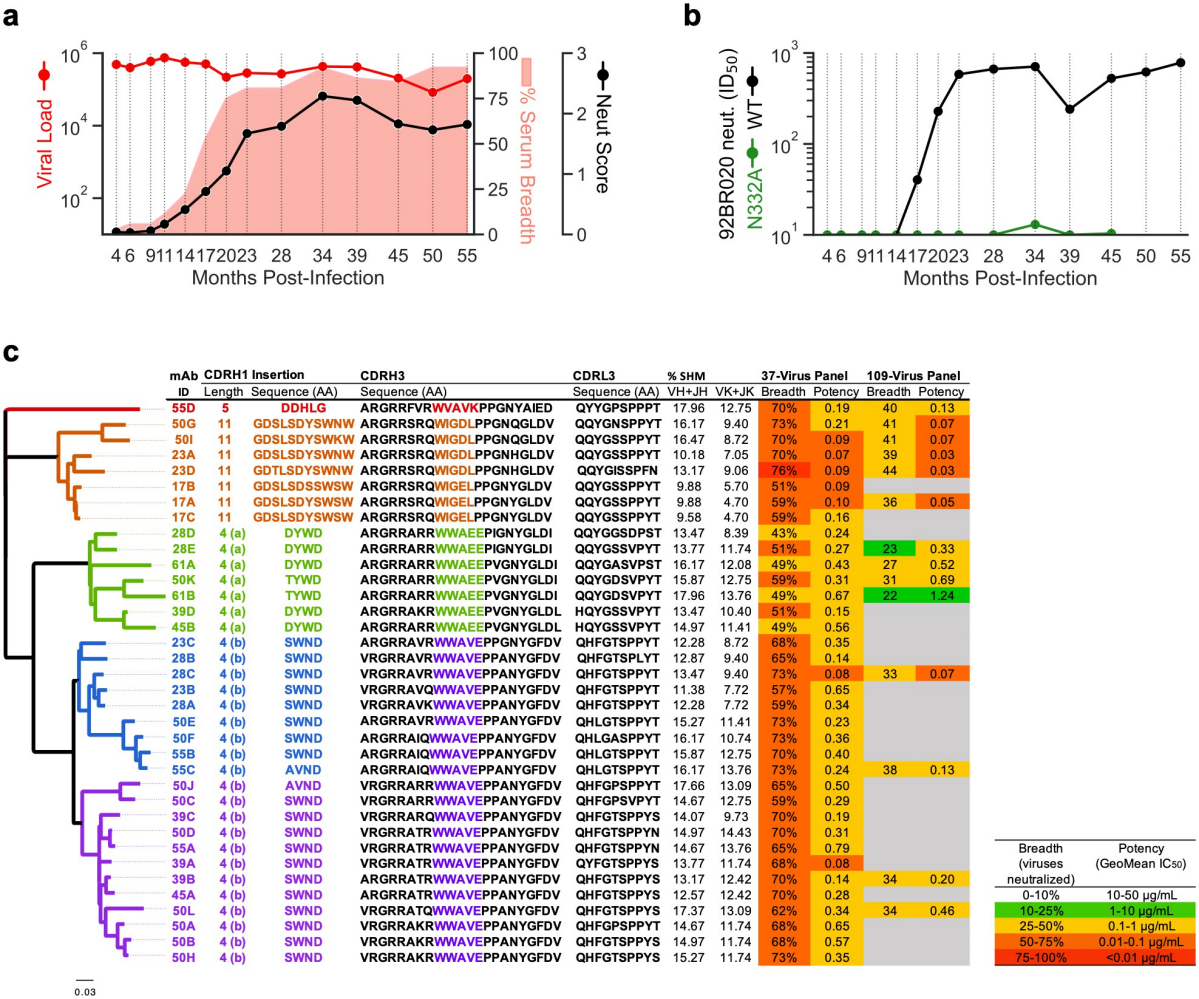
Development of a broadly neutralizing antibody response in donor PC39 and characteristics and phylogeny of PC39-1 bnAbs. **(a)** Longitudinal development (months) of heterologous serum neutralization. Serum neutralization breadth, defined as the % of viruses showing >50% inhibition of infectivity at the lowest plasma dilution of 1:50 using a 37-virus indicator panel, is shown in pink; neutralization score is shown as a black line and viral load as a red line. **(b)** Neutralization Inhibitory Dilution 50 (ID_50_) for a heterologous isolate, 92BR020, and an N332 knockout variant are shown in black and green, respectively, for each time point. **(c)** Characteristics of PC39-1 mAbs include insertion length relative to unmutated ancestor sequence, HC complementarity determining region 1 (CDRH1) insertion sequence, CDRH3 and CDRL3 sequences, HC and LC V- and J-gene nucleotide somatic mutation percentage, (%SHM), neutralization breadth and potency on two panels (colored as indicated; breadth is defined as viruses with IC_50_ < 50 μg/mL in TZM-bl pseudovirus assay, potency is defined as the geomean IC_50_ in μg/mL of neutralized viruses). Abs are organized and colored by clade identity based on CDRH1 insertion and named based on the time point (in mpi) from which they were isolated. The HC phylogeny for the PC39-1 mAbs is shown on the left and colored by antibody clade.

IAVI Protocol C is a large cohort of individuals in sub-Saharan Africa identified during primary HIV infection and then followed longitudinally, examining sera for the development of neutralization breadth [47]. Donor 39 (PC39), infected with a clade C virus and followed for more than 5 years, showed exceptional cross-clade neutralization breadth when tested against a global panel of 37 viruses (Fig 1A). The development of measurable breadth became clear at around 9–11 months post infection (mpi), expanded rapidly between 11 and 20 mpi and levelled off at >80% around 23 mpi. The potency of serum neutralization as reflected in a neutralization score [48] continued to increase out to around 34 months before levelling off (Fig 1A).

To determine the predominant broadly neutralizing specificities of the sera, we investigated neutralization of select variants of viruses from different clades. A greatly reduced ability for donor plasma to neutralize N332A Env-mutated pseudoviruses of different clades identified the V3-glycan bnAb site as the major target for PC39 neutralization breadth (Fig 1B) [49]. To isolate monoclonal antibodies (mAbs) targeting the V3-glycan site, we affinity sorted B cells from PMBCs by flow cytometry selecting positively for reactivity with 92BR020 clade B gp120 and negatively with the corresponding gp120 N332A variant (Fig 1B and S1(A) Fig). The use of a clade B gp120 given that the donor was infected with a clade C virus strongly favored the selection of bnAbs. In total, 36 clonally related mAbs were isolated across eight timepoints displaying varying levels of neutralization breadth (43–76%) and potency (0.08–0.67 μg/mL IC_50_) as measured on a 37-virus global panel (Fig 1C and S1(B) and S1(C) Fig). The bnAb lineage is defined by a CDRH3 length of 22 amino acids (aa), an IGHV4–34*01/IGHJ6*02 heavy chain (HC) and an IGKV3–20*01/IGKJ2*01 light chain (LC) gene (Fig 1C and S2 Fig). We termed this lineage PC39-1.

A striking feature of the PC39-1 lineage is the occurrence, in all isolated mAbs, of an insertion in the HC CDR1 (CDRH1) at position 31 (Fig 1C and S2 Fig). The majority of mAbs contained either an 11 (residues 31A-K) or a 4aa (residues 31A-D) insertion paired with a^100A^WIG(E/D)L^100E^ or ^100A^WWA(V/E)E^100E^ motif, respectively in the CDRH3, while only one isolated mAb had a 5aa insertion and a separate motif,^100A^WVAVK^100E^, in CDRH3. Two independent 4aa insertion events occurred within the developing lineage and can be found on two separate branches. The first 4aa insertion event (insertion 4a) resulted in a single branch predominantly defined by a ‘DYWD’ insertion in CDRH1 (there are two cases of a ‘TYWD’ insertion) and a^100A^WWAEE^100E^ motif in CDRH3. The second 4aa insertion event (insertion 4b) resulted in two branches both overwhelmingly defined by a ‘SWND’ insertion (two cases of ‘AVND’ and one of ‘IWND’) in CDRH1 and a^100A^WWAVE^100E^ motif in CDRH3.

The isolated mAbs alone did not fully recapitulate serum neutralization breadth, with the major difference being an inability to neutralize most CRF01_AE clade strains (S1(C) Fig and S3 Fig), likely attributable to the presence of a glycan at position 334 rather than 332 in this clade. Indeed, the only virus from this clade that was neutralized by PC39-1 mAbs retained a glycan at position 332 (S3 Fig).

In common with many V3-glycan-directed bnAbs, PC39-1 mAbs were dependent on the N301-glycan, and specific interactions with residues of the _324_GDIR_327_ motif at the base of V3, part of the co-receptor binding site, as well as a dependence on mutations in V1 (S4(A) Fig) [16, 23, 49–56]. Most mAbs were sensitive to D325 and R327 mutations, yet only those with the 11aa CDRH1 insertion were sensitive to mutation at every position in the _324_GDIR_327_ motif (S4(A) Fig). This difference extended to gp120 binding, with 11aa insertion variants displaying a higher affinity for gp120s as measured by SPR and an absolute dependence on N332 (S4(B) Fig). Interestingly, several mAbs with a 4aa insertion and the single mAb with a 5aa insertion retained some binding affinity to N332 glycan-deleted gp120s as measured by ELISA, despite their dependence on N332 for neutralization.

### NGS investigation of the bnAb lineage

**Fig 2:**
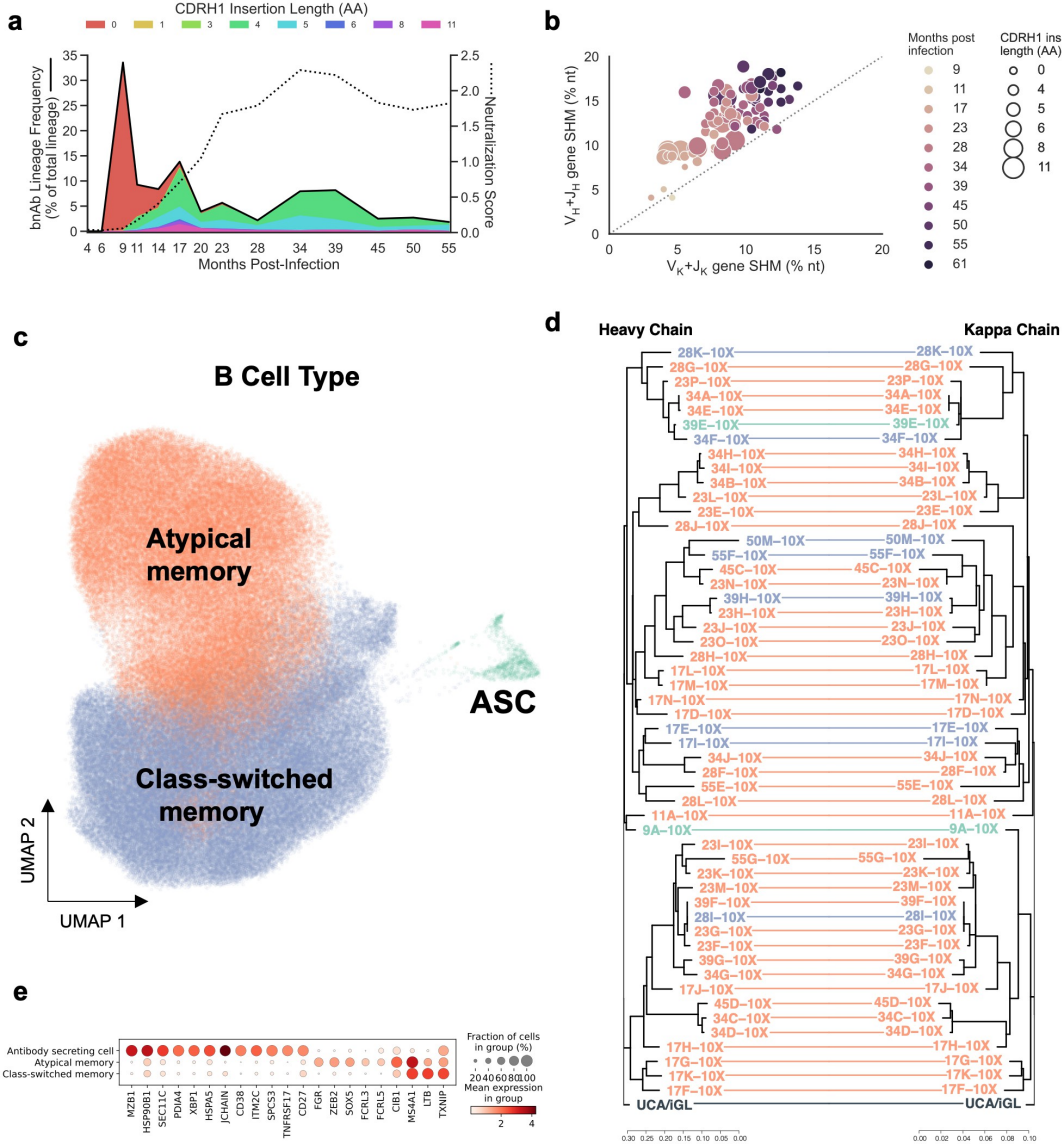
PC39 antibody next generation sequencing. **(a)** The frequency of PC39-1 bnAb lineage transcripts identified from bulk heavy-chain NGS is shown longitudinally, as a percentage of total heavy chain transcripts (black line) with the contribution of each CDRH1 insertion length shaded in underneath and colored as indicated. Serum 37-virus panel neutralization score is shown as a dotted line. **(b)** Scatterplot of PC39-1 bnAb lineage mAbs isolated from both FACS and 10X HC (V_H_+J_H_) vs LC (V_K_+J_K_) somatic hypermutation (SHM, % nucleotide), colored by months-post infection of mAb isolation. The size of each dot indicates CDRH1 amino-acid insertion length. **(c)** UMAP projection of circulating memory B cell scRNA-seq clusters (198,294 cells; twelve time-points). **(d)** Tanglegram of corresponding HC (left) and LC (right) phylogenies for the PC39-1 bnAb lineage members isolated via 10X, colored by transcriptional phenotype as indicated. **(e)** Mean marker gene expression for B cell subsets. Dot size depicts the frequency of cells in which a gene is detected.

To investigate lineage maturation in more detail, we performed longitudinal bulk next-generation sequencing (NGS) of antibody heavy-chain transcripts from purified peripheral IgG+ memory B cells. The frequency of PC39-1 lineage transcripts over time revealed a large peak of expansion at 9 mpi followed by a rapid contraction concomitant with increased broadly neutralizing plasma activity (Fig 2A), a phenomenon that has been described for prior protocol C donors [21, 23, 24, 27]. CDRH1 insertions could be detected as early as 11 mpi, and their frequencies in the PC39-1 lineage increased to completely dominate by month 55 with the two sublineages having a 4aa insertion being much more abundant than the sublineage with an 11aa insertion (Fig 2A). However, the presence of nearly every length of insertion from 1–11 amino acids (S5(B) Fig shows 4, 5 and 11 aa insertions but insertions of length 1 (1 clones), 3 (5 clones), 6 (4 clones) and 8 (15 clones) aa were noted) could be found in the NGS HCs and were the result of adjacent sequence duplication. Somatic hypermutation within the lineage was found to increase over all time-points (Fig 2B and S5(C) Fig).

The earliest Ab heavy chains from the lineage were detected at 9 mpi in the bulk HC NGS dataset, including a sequence with 99.4% identity to VH4-34*01/JH6*02 and lacking any CDRH1 insertion (S2 Fig). Reverting the single mutation in this HC, combined with a reverted germline LC sequence (iGL, see Methods), gave us an unmutated common ancestor (UCA) (S2 Fig). This led us to hypothesize that the lineage began at or near 9 mpi due to the observed peak (Fig 2A) and we reasoned that it should be possible to isolate natively paired unmutated or near-germline members of the antibody lineage. However, our attempts to use flow-cytometry to isolate memory B cells at 9 mpi using the approach described above were unsuccessful and we turned to an alternate approach.

### Isolation of early lineage precursors using the 10X Genomics single cell platform

The first attempt using the 10X platform yielded one HC-LC paired bnAb precursor (4.1% HC SHM and 4.6% KC SHM, no indels) at 9 mpi. We continued the methodology on the remaining time-points to isolate 52 mAbs of various CDRH1 insertion lengths (Fig 2B–2D). Monoclonal Abs isolated via 10X were found on the same phylogenetic branches as the mAbs isolated via flow-cytometry as well as new branches of the lineage that had only been previously sampled from bulk HC-only NGS (S5(A) and S5(B) Fig). A comparison of lineage frequency over time between bulk HC NGS and single-cell sequencing showed that the peak at 9 mpi in the NGS data was absent in the single cell data (S5(D) Fig). Gene expression analysis of 198,294 circulating memory B cells across 12 time-points identified three main B cell phenotypes (class-switched memory, atypical memory and antibody-secreting) that were defined based on preliminary annotation with CellTypist [57] as well as marker genes (Fig 2C–2E and S6 and S7 Figs). This allowed us to speculate that the discrepancy between the lineage frequency based on bulk vs. single-cell methods was due to lineage plasmablast expansion at 9 mpi that was not present in the remaining time-points (Fig 2C and 2D, S5(D) and S7(D) and S7(E) Figs). Plasmablasts with their high levels of antibody mRNA will greatly bias the bulk NGS lineage frequency but not that detected by single cell methods. Intriguingly, most lineage members were shown to have an atypical memory phenotype (42/52) followed by class-switched memory (8/52), and antibody-secreting (2/52). The atypical memory phenotype has been associated with chronic antigen exposure [58, 59] and may suggest that continuous B cell engagement by Env contributed here to the development of breadth in the PC39-1 lineage.

Since the VH4-34 HC has been associated with self-reactivity [60–65] and since some of the mAbs isolated were arginine rich in the N-terminal region of the CDRH3, a feature associated with DNA reactivity [66], we investigated poly- and self-reactivity of a selection of PC39-1 lineage Abs. As shown in S8(A) and S8(B) Fig, with one or two exceptions, there was little evidence for any self- or polyreactivity. The UCA showed no reactivity with glycans on a NCFG microarray v5.5 [67] (S8(C) Fig), arguing that the lineage did not arise from an anti-glycan antibody, for example elicited against a glycosylated parasitic or bacterial antigen that then developed specificity for the protein part of its epitope on HIV Env.

### Viral evolution and the initiation of the bnAb lineage

**Fig 3:**
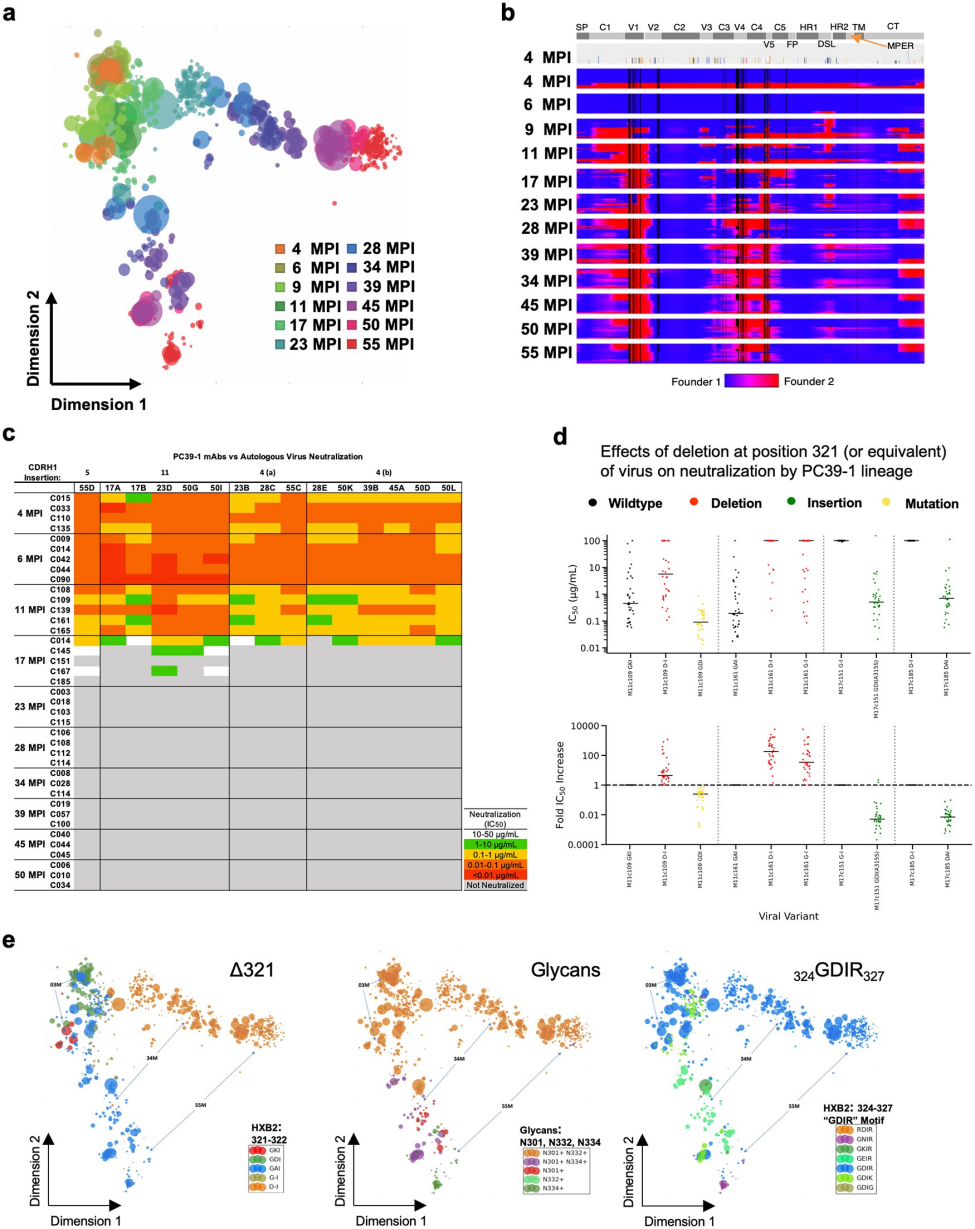
PC39 virus evolution and escape from bnAb lineage. **(a)** Multidimensional scaling plot of PC39 virus sequences, colored by time point as shown. **(b)** Recombination plot representing individual *env* high-quality consensus-sequences (HQCSs) from PacBio sequencing as lines by time point, with each residue location colored by the most closely related founder variant as indicated. The top block shows amino acid positions that vary between the two founder variants in colors. **(c)** Matrix of IC_50_ neutralization of autologous clones from PC39 at the given time point in mpi (rows) for PC39-1 mAbs (columns), colored as indicated. **(d)** IC_50_ (top) and fold change (bottom) for PC39-1 Ab neutralization of the indicated autologous clones and the given mutations, colored according to the type of mutation. A three-letter code is used to designate residues from HXB2 positions 321 to 322 (HXB2 itself has a deletion phenotype, so has only 2 amino acids in the equivalent region), and a two-letter code in the mutant denotes a deletion at any position in the triad. **(e)** Multi-dimensional scaling plot of PC39 Env populations, colored as indicated by various motifs, with time mpi indicated by arrows.

Next, we sought to learn more about the virus(es) that had infected donor PC39 and their evolution through the course of infection. Viral sequencing and phylogenetic analysis suggested that two founder variants were involved in early infection. The variants differed by 6% under the GTR evolutionary model (S9(A) Fig). Due to founder variant recombination confounding further phylogenetic analysis, multidimensional scaling of viral sequences was employed to further investigate viral evolution. This analysis revealed a striking pattern of mixing between founders before subsequent independent evolution at around 17 mpi (Fig 3A and 3B). Investigation of sequences in the individual believed to have infected the PC39 donor revealed a diverse population of viruses that included related variants to the two founder variants (S9(B) Fig). We concluded that the epidemiologically linked yet substantially divergent founder variants are related, and that the source individual probably infected the donor with the two variants either at the same time or during a limited time-period.

Understanding in as much detail as possible the HIV Envs that triggered which bnAb lineages and how the triggering occurred could be valuable for HIV vaccine design [11–13]. The PC39-1 lineage results offer some potential insights. Early bnAb precursors are well defined in this case as are the predominant viruses around the time of the emergence of the lineage. Early bnAb precursors isolated via 10X that lacked insertions (Fig 2) were synthesized, expressed and tested for binding by SPR to founder variant gp120 and NFL trimer proteins. No reactivity was found (S10(A) and S10(B) Fig). The bnAb precursors were then tested for neutralization of autologous viruses and showed no activity (S10(C) Fig). Similar findings were made for a computationally inferred PC39-1 UCA (S10(D) Fig). The data suggest that Env molecules in the case of infection and potentially clustered on a membrane may be able to trigger bnAb lineages at lower effective affinity for bnAb precursors than recombinant vaccine proteins, for which relatively high affinities may be required [68].

### Neutralization escape from the bnAb lineage

Eventually virus escapes from bnAbs of the PC39-1 lineage and this occurs relatively precipitously at around 17 mpi (Fig 3C). To identify the mutations responsible for escape, we selected sensitive and resistant viral envelopes from time-points before and after escape (S11(A)–S11(C) Fig). Specifically, we included mutations that differed between our selected sensitive and resistant variants, as well as mutations occurring at any point in the autologous population that are known to affect V3-glycan Ab neutralization. Most strikingly, a deletion at 321 fully explained the difference in neutralization between the selected variants (S11(A)–S11(C) Fig). One hypothesis to explain the effect of this deletion is that it recesses the GDIR portion of the V3 loop, preventing contact with the antibody. Furthermore, reversion of the deletion in two early resistant viruses restores neutralization by PC39-1 Abs (Fig 3D and S11(D) Fig), confirming the importance of the deletion for neutralization escape.

Analysis of the prevalence of the deletion phenotype in the autologous viral population led to the observation that the Δ321 deletion variant is maintained in a single “arm” of the viral escape lineage but does not persist past 34 mpi in the other (Fig 3E). We looked for canonical V3-glycan resistance mutations in the lineage, and found that _324_GDIR_327_, N301 and N332 mutations occurred together in the other “arm” of virus evolution (Fig 3E). This pattern has been observed in other studies; _324_GDIR_327_ residues are implicated in CCR5 co-receptor binding and the loss of the camouflaging glycans at N332 and N301 results in greater selection pressure on the GDIR motif [56].

After viral escape, PC39-1 Abs continue to evolve to generate greater breadth of neutralization as assessed in the neutralization score out to at least 34 mpi (Fig 2A–2C and S2–S5 Figs). There is some decline in the neutralization score between 35 mpi and 55 mpi but this is small and may not be significant. One hypothesis to explain the observation of increased neutralization score after escape is continued affinity maturation in long-lived GCs [69].

### Structural consequences of the CDRH1 insertion

Since the development of neutralization breadth was associated with an insertion of varying length at CDRH1, we sought to investigate the consequences of the insertion on antibody structure. X-ray crystal structures were solved for three 11aa-insertion Fabs (17A, 23D and 50I) and three 4 aa-insertion (group 4b) Fabs (50E, 55C and 50L) and the PC39-1 lineage UCA Fab. Each PC39-1 mAb is named after the timepoint (in mpi) at which it was isolated.

**Fig 4:**
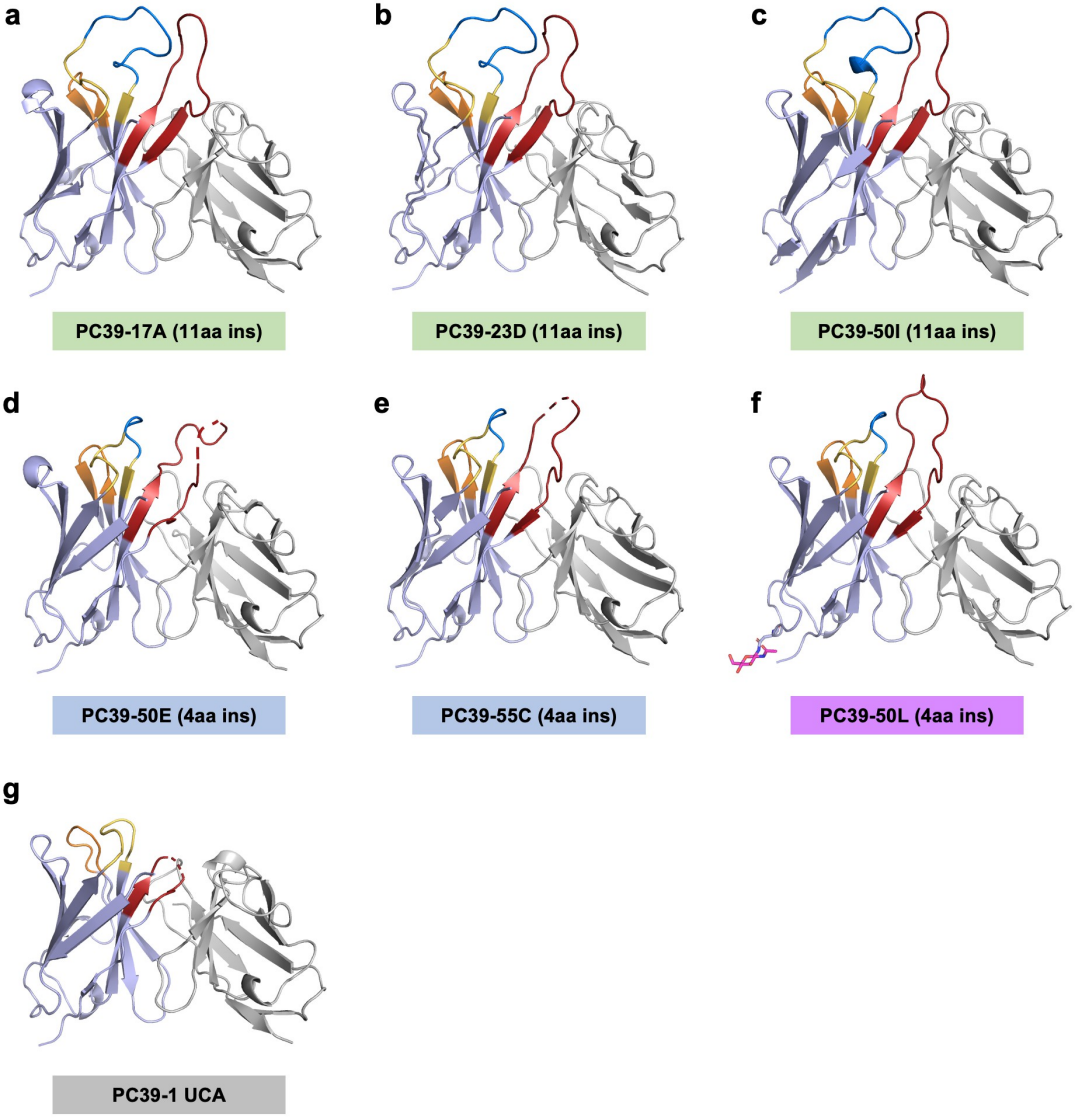
X-ray structures of PC39-1 Fabs. **(a)** Ribbon representation of PC39-17A with 11aa CDRH1 insertion. HC CDRs are colored (CDRH1 in yellow, CDRH2 in orange, CDRH3 in red) and the 11aa insertion in CDRH1 shown in blue. Other regions of the HC are in lavender and the LC in grey in all panels. **(b)** Ribbon representation of PC39-23D Fab with 11aa CDRH1 insertion. **(c)** Ribbon representation of PC39-50I Fab with 11aa CDRH1 insertion. **(d)** Ribbon representation of PC39-50E Fab with 4aa CDRH1 insertion. **(e)** Ribbon representation of PC39-55C with 4aa CDRH1 insertion. **(f)** Ribbon representation of PC39-50L with 4aa CDRH1 insertion. **(g)** Ribbon representation of PC39-1 UCA with no insertion in CDRH1.

Structures of all three 11aa-insertion Fabs reveal an extended CDRH1 that provides support to and stabilizes the long 22aa CDRH3 (Kabat numbering; Fig 4A–4C and S12 Fig) of the lineage. The similarity in backbone conformation (root-mean-square deviation (RMSD) = 0.3Å) and neutralization potency (0.10 vs. 0.09 respectively) of mAbs 17A and 50I suggests that the preconfigured interface in these structures is conserved across 33 months of Ab maturation. However, neutralization breadth was shown to increase during this time highlighting the importance of continued SHM (S3 Fig). The glycine at position G_100C_ found in the WIGEL motif is present in the UCA and only retained in 11aa-insertion Abs (S2 Fig). This sequence provides a glycine hinge to the unique mature bnAb CDRH3 structure.

In contrast to the 11aa-insertion Fabs, structures of 4aa-insertion mAbs 50E and 55C revealed a more flexible and more difficult to resolve CDRH3 (Fig 4D and 4E). However, we were able to fully resolve the CDRH3 of another 4aa-insertion mAb 50L and show that CDRH1 participates in stacking interactions to stabilize the CDRH3 despite the shorter insertion length (Fig 4F). These differences in CDRH3 stability between the two antibody clades might explain contrasting neutralization potency despite similar breadth (Fig 1C).

When compared to other V3-glycan bnAb Fab structures, 11aa-insertion mAb HCs (17A, 23D and 50I) most closely resemble PGT135, which also has an insertion in CDRH1, while the LCs of these mAbs are more similar to that of PCDN-38B [23], with an RMSD of 0.48 Å (S12(A) Fig). The 4aa-insertion Fabs 50E and 55C could not be as readily compared to previously described N332-dependent bnAbs due to their CDRH3s, which are not completely resolved. Intriguingly, the 4aa-insertion Fab 50L differs substantially in structure from 11aa-insertion Fabs as well as from other V3-glycan Fabs due to a slightly more elongated conformation of its CDRH3 (Fig 4 and S12(A) Fig). A triad of Trp residues are located in the binding site of these antibodies; Trp residues are present in both of the CDRH1 insertions (11 and 4aa groups), but do not appear to be close enough to directly interact with the Trp residues found in either CDRH3 motif (^100A^WIG(E/D)L^100E^ or^100A^WWAVE^100E^) (S12(B) Fig).

## Discussion

We describe here an HIV infected individual who developed serum antibody neutralization breadth incorporating several interesting features that may shed light on strategies for bnAb-targeted vaccine design.

The infection course of donor PC39 was unusual in that two distinct but related founder viruses were established early in the individual. The two viruses either infected the individual in a single transmission event or from repeated exposure to the infecting partner early in the infection. The related founder viruses then underwent extensive recombination, and this occurred concomitant with the initiation and development of neutralization breadth. The development of breadth under these circumstances is consistent with observations of a higher rate of neutralization breadth in superinfection in some cases although not others [18, 25, 38–42] and of the association of neutralization breadth with multiple founder viruses [43]. The latter is particularly comparable to the current case and lends some support to the observations of the authors of that previous study and their suggestion for an HIV vaccine strategy: “These results demonstrate that the presence of slightly different HIV-1 variants in acute infection could promote the induction of bnAbs, suggesting a novel vaccine strategy, whereby an initial immunization with a cocktail of minimally distant antigens would be able to initiate bnAb development towards breadth”. Having undergone extensive recombination, two virus arms then emerged at around 17 months and these two arms showed distinct features including the details of neutralization escape.

In our longitudinal study, neutralization escape occurred rapidly once the PC39-1 bnAb lineage emerged, but the lineage continued to develop in terms of neutralization potency for several months. This is consistent with ongoing affinity maturation in long-lived germinal centers (GCs) as has been described [69]. It is tempting to speculate that the sequestration of multiple related viruses on FDCs in GCs might favor the maturation of bnAbs, for example by enhancing internalization of antigen that could be achieved relative to nAbs with narrower specificities.

The initiation of neutralization breadth coincided with the appearance of indels in the CDRH1 of PC39-1 lineage Abs. A series of independent insertion events occurred at the same position in CDRH1 to yield clones with insertions of lengths varying from 1–11 amino acids. The predominant insertion lengths were 4 and 11 amino acids with the former arising from two independent events and showing different characteristics. The structural importance of the 11 amino acid CDRH1 insert appears to be to stabilize the long CDRH3 of the PC39-1 lineage Abs. For the 4 amino acid CDRH1 insert, stabilization is less apparent. However, in both 4 and 11 amino acid cases, the acquisition of a triad of tryptophan residues from CDRH1 and CDRH3 is observed, but it is not yet clear what the functional and evolutionary consequences are of this particular feature. HIV immunogen design based on insights gained from bnAbs (reverse vaccinology 2.0 [3, 8]) has tended to prefer bnAbs without insertions or deletions (indels) with the understanding that this requirement may increase the difficulty of inducing bnAbs in a sequential immunization strategy [13]. The frequency of insertions in the IgM memory and IgG memory of healthy individuals is of the order of 2% [44–46] with a decreasing frequency as the insertion length increases (S13 Fig). The demonstration here of multiple independent insertions at a sensitive site suggests that there may be opportunities for the generation of such insertions if an appropriate antigen stimulation via vaccination can be achieved. Given that V3-glycan bnAbs interact with and/or accommodate glycans, there was a formal possibility that the PC39-1 lineage arose from an initial clone generated against a glycan moiety. Our study showed no evidence for glycan reactivity in lineage precursors. Another concern that arose was that the PC39-1 VH gene segment used is VH4-34, which has been associated with self-reactivity. However, there was little evidence for self-reactivity of the great majority of PC39-1 lineage members. Therefore, it seems most probable that the lineage was triggered by the Env antigen itself.

Overall, this study, together with another recent study [43], suggests a potential advantage for inducing bnAbs in an immunization strategy including multiple related Env (MRE) molecules. Of note, the studies describe the development of breadth after exposure to multiple founder viruses, and their recombinants in our case, that are closely related but not as similar generally as the swarm of viruses predominating at any one-time during cases of acute infection with a single virus. Indeed, Keele et al. concluded, from a cohort of 102 subjects with acute HIV infection, that individuals infected with a single virus showed *env* diversity ranging from 0.08 to 0.47% while individuals infected with multiple related viral variants from a single source partner (either in the same transmission event or multiple transmission events over time) showed *env* diversity ranging from 0.86 to 6.63% [70]. Therefore the *env* diversity of PC39 at 6% at early time-points is in the upper range of previously reported numbers for multiple related variant infections. Interestingly, Piantadosi et. al found a positive correlation (Spearman’s rho = 0.51, P = 0.008) between *env* diversity at acute infection and nAb breadth at 5 years post-infection in a cohort of 26 non-superinfected individuals who had a median *env* breadth of 0.28% (range, 0 to 4.0%) [71]. However no association was found between *gag* diversity at acute infection and nAb breadth at 5 years post-infection. This study again supports a possible role for early encounter with MRE in the development of neutralization breadth.

It should be noted however that, in order to make the claim that increased serum nAb breadth in donors infected with multiple related viruses is due to true bnAb development and not a polyclonal response consisting of multiple isolate specific nAbs, targeting of bnAb epitopes must be shown. Futhermore, the nAb response elicited by different founder viruses should be shown to be the same. Both demonstrations were made above for PC39 bnAbs. Still, questions remain—is PC39 bnAb development due to a prime-boost in which the individual was infected with two variants over multiple time-points or is it due to a cocktail effect in which the bnAbs develop against MRE molecules in a single germinal center? Or both? The development of bnAb breadth as the result of BCRs reacting with a cocktail of recombinant viruses seen between 9 and 17mpi is consistent with initiation of the lineage at 9mpi and viral escape at 17mpi.

A potential limitation of our study is the lack of available sequences from acute infection. As a consequence, we are unable to clearly define the true founder viruses from donor PC39. However, although advanced techniques for inferring founder multiplicity exist [72], our study has convincingly demonstrated that the initial variants sequenced from donor PC39 exhibit a close genetic relationship to different, distinct variants identified within the diverse source donor population (as illustrated in S9(B) Fig). Therefore, despite this caveat, our findings strongly support the idea that the PC39-1 bnAb lineage is not a consequence of superinfection, but rather the outcome of interactions with MRE from related founders.

In contrast to infection with multiple closely related founder viruses, superinfection typically shows greater sequence differences between infecting viruses and has not been clearly associated with the development of neutralization breadth [18, 25, 38–42]. Within a given host, HIV *env* diversity in cases of superinfection can range from 10% (for inter-subtype superinfection) to >30% (for intra-subtype superinfection) [73]. Therefore, there is a suggestion that there may be a “sweet spot” in terms of the relatedness of immunogens in a cocktail to favor the development of breadth. Indeed, modeling studies [74–76] have focused on the importance of mutational distance between antigens in a putative cocktail. The authors have suggested that, when variant antigens are separated by only small mutational differences, then strain specific antibodies develop. When variant antigens are too different then affinity maturation cannot be sustained and few antibodies result.

Finally, in terms of activating appropriate bnAb precursors, there is a concern about the frequency of such precursors in the naïve B cell repertoire so that immunization will be effective in the overwhelming majority of vaccinees [77–79]. This concern may not be well addressed by the MRE strategy. However, if appropriate germline activating immunogens are designed [30, 34–36] then the MRE strategy may be worthy of investigation as a boosting step in a sequential immunization protocol.

## Materials and methods

### Ethics statement

Serum and PBMC samples were obtained from donor PC39 enrolled in the IAVI Protocol C longitudinal HIV-1 infection cohort. IAVI protocol C enrolled 613 individuals from sub-Saharan Africa who were rapidly screened for HIV Abs after recent exposure to HIV-1. Samples were collected with written, informed consent, and clinical protocols were approved by the Republic of Rwanda National Ethics Committee, Emory University Institutional Review Board, University of Zambia Research Ethics Committee, Charing Cross Research Ethics Committee, UVRI Science and Ethics Committee, Kenyatta National Hospital Ethics and Research Committee, KEMRI Scientific and Ethics Review Unit, University of Cape Town Research Ethics Committee, University of Kwazulu-Natal Biomedical Research Ethics Committee, Mahidol University Ethics Committee, Veterans Affairs San Diego Institutional Review Board, and Scripps Institutional Review Board.

### Pseudovirus production and neutralization assays

Neutralization assays were performed by incubating Abs or sera with HIV-1 pseudoviruses that are capable of one round of infection to TZM-BL cells as described [80]. Pseudoviruses were produced in HEK293T cells unless those specifically indicated as made in HEK293S cells [81]. Cells were transfected with a full-length Env expressing plasmid and an Env-deficient genomic backbone plasmid (pSG3ΔEnv), cultured for 72 hrs before use. Kifunensine and swainsonine were applied to HEK293T cells on the day of transfection at 25 and 20 μM concentrations, respectively [82]. Serum samples were heat-inactivated at 56°C for 45 min prior to use. Monogram Biosciences cloned autologous viruses from serum and tested the PC39 mAbs for neutralization using methods previously described [83]. Full-length autologous *env* gene strings were synthesized for representative clones of each time point using GeneArt gene synthesis (Life Technologies), then cloned into pcDNA3.1 vector (Life Technologies) for pseudovirus production. Mutagenesis was performed using Quikchange site-directed mutagenesis kit (Agilent Technologies).

### Serum depletion

Tosylactivated Dynabeads (Life Technologies) were conjugated with Bovine Serum Albumin (BSA), or WT and N332A 92BR020 rgp120 proteins. Serum samples were depleted of Abs binding to these proteins through multiple rounds of immunoprecipitation as previously described [84]. The depletion of Abs of the desired specificity was confirmed by ELISA prior to using depleted serum in pseudovirus neutralization assays.

### Single memory B cell sorting and Ab isolation

Sorting of antigen- and epitope-specific memory B cells was performed as previously described [85–87]. Single antigen-specific (gp120 WT positive gp120-N332A negative) IgG+ B cells were sorted into wells of 96-well PCR plates. RT-PCR was performed using multiplex PCR methods and primers, then cloned into heavy or light chain constant region encoding vectors as described by Tiller et al [86]. After size verification of the PCR product, DNA was sequenced, and V, D and J genes were assigned using IMGT/V-quest for human Ig analysis [88, 89] (http://www.imgt.org/IMGT_vquest/). Individual Ab HC and LC sequences are available from GenBank database (accession numbers: OQ747680—OQ747752).

### Monoclonal antibody production and purification

Antibodies and Fab fragments were produced with plasmids encoding full-length or truncated HC (after constant region 1, CH1) HC and full-length LC. For large-scale production, HC constructs and LC were transiently expressed with the FreeStyle 293 Expression System (Invitrogen). Supernatant was collected after 4 days of culture and whole IgGs were purified with Protein A Sepharose (GE Healthcare) followed by buffer exchange to 1X PBS.

### ELISA binding assays

Half-area 96-well ELISA plates were coated overnight at 4°C with 50 μL PBS containing rgp120 at 20 μg/ml. The wells were extensively washed with PBS 0.05% Tween 20 and blocked with 3% BSA. Serial dilutions of sera or Ab were then added to the wells, and the plates were incubated at room temperature for 1 hour. After washing, goat anti-human IgG F(ab’)2 conjugated to alkaline phosphatase (Pierce), diluted 1:1000 in PBS containing 1% BSA and 0.025% Tween 20, was added to the wells. The plates were incubated at room temperature for 1 h, washed, and developed by adding alkaline phosphatase substrate (Sigma) according to the manufacturer’s instructions. The optical density at 405 nm was read on a microplate reader (Molecular Devices). EC_50_ values were calculated using Prism6 (GraphPad).

### Bio-layer interferometry (BLI) binding analysis

To obtain binding curves by BLI, we used an Octet Red instrument immobilizing IgGs on previously hydrated (PBS pH 7.4) anti-human IgG Fc sensors (Fortebio, Inc.). The gp120 were analyzed as analytes free in solution (PBS pH 7.4). Briefly, the bio-sensors were immersed in PBS pH 7.4 containing IgGs at 10 μg/mL for 1 minutes and 1000 rpm prior to encounter the analyte (gp120 at 1 μM). The IgG-immobilized sensor was in contact with the analyte in solution for 5 minutes at 1000 rpm and then removed from the analyte and placed into PBS pH 7.4 for another 10 minutes. These intervals generated association and dissociation binding curves after subtraction of binding signal to a negative control IgG.

### Bulk Ab heavy-chain next-generation sequencing (NGS) and pre-processing

RNA was prepared (RNEasy kit, Qiagen) either from whole PBMC or IgG+ memory B cells separated from approximately 1 x 10^6^ PBMCs by selective depletion (Switched memory B cell isolation kit; Miltenyi Biotec). RNA was subjected to RT-PCR using barcoding primers that contain unique molecular identifiers (UMIs) as previously described [90, 91]. cDNA was then amplified using primers previously described [92]. Illumina sequencing adapters and sample-specific indexes were added during a second round of PCR. Samples were quantified using fluorometry (Qubit; Life Technologies), pooled at approximately equimolar concentrations, and the sample pool was re-quantified to be loaded onto an Illumina MiSeq. Paired-end MiSeq reads were merged with PANDAseq [93]. Merged reads were collapsed and error corrected from UMI sequences via AbCorrect (https://github.com/briney/abtools#abcorrect). Raw fastq files are available on the SRA database (BioProject PRJNA943240).

### Single-cell RNA and VDJ sequencing with 10X Genomics and raw data pre-processing

Memory B cells separated from approximately 1 x 10^6^ PBMCs by selective depletion (Switched memory B cell isolation kit; Miltenyi Biotec) were loaded according to the 10X Genomics manufacturer’s protocol for 5’-gene expression to attain 10,000 sequenced cells per well. Gene expression and VDJ libraries were prepared according to manufacturer’s protocol before sequencing on either an Illumina NextSeq or NovaSeq. BaseCall files were subsequently used to generate library-specific FASTQ files with cellranger mkfastq (v7.0.0) prior to running cellranger count (v7.0.0) with the GRCh38 (release 92) reference to produce cell barcode-gene expression matrices using default settings. For single-cell VDJ datasets, cellranger vdj (v7.0.0) was run using the refdata-cellranger-vdj-GRCm38-alts-ensembl-7.0.0.tar.gz reference from 10x Genomics using default settings. Raw fastq files are available on the SRA database (BioProject PRJNA943240). Individual 10x Genomics-derived PC39-1 Ab HC and LC sequences are available from GenBank database (accession numbers: OQ747575—OQ747679). Gene expression data is available at GEO (accession number: GSE229232).

### Integrated bulk Ab heavy-chain and single-cell VDJ sequence analysis

All single-cell VDJ sequences were combined with FACS-derived mAb heavy-chain sequences and pre-processed bulk Ab heavy-chain sequences from the donor PC39. Ab germ-line assignment, junction identification and other basic Ab information was determined using AbStar [94]. Sequences were assigned to clonal lineages using Clonify, a software package specifically developed for Ab lineage assignment [92]. Bulk Ab NGS-derived PC39-1 lineage sequences were clustered at 97.5% identity with VSEARCH [95], singletons removed, and the size of each cluster was recorded. Bulk Ab NGS cluster centroids combined with scVDJ and FACS-derived PC39-1 heavy-chain sequences were used to generate a multiple sequence alignment with with MAFFT [96], and a tree file was calculated with FastTree using default settings [97]. The phylogenetic tree was drawn in Python using the ETE Toolkit [98]. The inferred germline (iGL) light chain sequence was determined by reverting the least mutated light chain found amongst mAbs directly to GL. The tanglegram of 10X scVDJ derived Ab pairs was made in R with the dendextend package [99].

### Single-cell RNA-seq data quality control, processing, and annotation of B cell clusters

Gene expression count matrices from Cell Ranger were used to calculate quality control metrics with Scanpy [100]. scanpy.pp.calculate_qc_metrics was used to calculate percentage mitochondrial expression per cell barcode and percentage ribosomal gene expression per cell barcode. Doublet detection was performed for each sample using the Scrublet algorithm as previously described [101] with scanpy.external.pp.scrublet, and predicted cell doublets were removed. V, D and J gene counts for immunoglobulin and T cell receptors were removed from the gene expression matrices. Immunoglobulin constant region genes (IgG1–4, IgA1-A2, IgL1–7 and IgK) were summed together and similarly removed. The modified gene expression matrices were then used for the removal of genes expressed in fewer than 3 cells. Cell barcodes with <200 genes detected were removed, as were cell barcodes with >10% mitochondrial reads or <4% ribosomal genes. Gene expression matrices were normalized by a factor of 10,000 and log transformed. CellTypist [57] was used for the initial prediction of cell types with the Immune_All_High model, and any cell predicted to be a non-B cell or naïve-B cell was removed. CellTypist was again used for the prediction of B cell types with the Immune_All_Low, COVID19_Immune_Landscape and Healthy_COVID19_PBMC models, and again any cell predicted to be a non-B cell was removed. Gene-expression matrices were converted back to raw count data and the top 4,000 highly variable genes were calculated using scanpy.pp.highly_variable_genes with the ‘seurat_v3’ flavor. Gene expression matrices were again normalised by a factor of 10,000 (scanpy.pp.normalize_per_cell) and log transformed (scanpy.pp.log1p) before subsetting the gene expression matrix to the calculated top 4,000 expressed highly variable genes. Data feature scaling (scanpy.pp.scale) and PCA (scanpy.tl.pca) was done. Neighborhood graph computation (scanpy.pp.neighbors) using n_pcs = 30 and n_neighbors = 20 was performed before embedding the neighborhood graph with Uniform Manifold Approximation and Projection (UMAP) [102] with scanpy.tl.umap. The Louvain method for community detection was used to cluster the cells (scanpy.tl.louvain). Batch correction was performed with the BBKN package [103] via bbknn.bbknn by treating each initial PBMC sample as a batch. Differential gene expression was calculated between cell clusters with scanpy.tl.rank_genes_groups using the Wilcoxon rank sum method. A combination of CellTypist predicted labels and examination of known cell marker genes was used for the identification of cell types.

### Glycan microarray analysis

PC39-1 UCA was screened on a printed glycan microarray, version 5.5, from the Consortium for Functional Glycomics (CFG) as described previously [67]. Antibodies were used at 30 μg/mL and were precomplexed with 15 μg/mL secondary antibody (goat anti-human-Fc-rPE, Jackson Immunoresearch) before addition to the slide. Complete glycan microarray can be found at https://ncfg.hms.harvard.edu/microarrays.

### Production of PC39 Fabs

Fab fragments of the respective Abs (PC39 UCA, 17A, 23D, 50C, 50E, 50I, 50L and 55C) were expressed in FreeStyle 293F cells (Invitrogen), at a 2:1 ratio of heavy to light chain and harvested 5–6 days after transfection. Fragments were purified by affinity chromatography (anti-human kappa—GE) and cation exchange chromatography (Mono S 10/100 GL), as previously described [104]. Purified fractions were analyzed by gel electrophoresis, concentrated, and subjected to crystallization trials with the IAVI/JCSG/TSRI CrystalMation robotic system (Rigaku) at 20°C, either in the cation exchange elution buffer, or after exchange into 20 mM Tris, 150 mM NaCl, pH 7.4 (Fab 17A).

### Crystallization and data collection

Crystals of PC39-17A at 14 mg/mL were obtained from a condition containing 0.2 M calcium acetate, 0.1 M imidazole pH 8.0, and 10% polyethylene glycol 8000. The crystals were cryoprotected in the reservoir solution and 20% ethylene glycol via brief immersion prior to vitrification in liquid nitrogen. PC39-23D crystallized at 14 mg/mL in 0.1 M HEPES pH 7.5 and 10% polyethylene glycol 8000, and was cryoprotected with reservoir solution and 10% ethylene glycol. PC39-50I Fab at 13.7 mg/mL crystallized in 10% glycerol, 0.1 M MES pH 6.0, 5% polyethylene glycol 1000, and 30% polyethylene glycol 600, and 50L at 10.1 mg/mL in 0.085 M sodium acetate pH 4.6, 0.17 M ammonium acetate, 15% glycerol, and 25.5% polyethylene glycol 4000—neither required further cryoprotection prior to plunging into liquid nitrogen. PC39-50E crystallized at 7 mg/mL in 0.2 M sodium formate and 20% polyethylene glycol 3350, and was cryoprotected in reservoir solution with 30% glycerol added before freezing. PC39-55C crystallized at 7.5 mg/mL in 0.2 M ammonium fluoride, 15% ethylene glycol, and 20% polyethylene glycol 3350. 55C also did not require further cryoprotection. The PC39-1 UCA crystallized in 0.2 M sodium chloride, 0.1 M CAPS pH 10.5, 20% polyethylene glycol 8000 at 10.6 mg/mL and was cryoprotected with reservoir solution and 20% glycerol. Diffraction data for four antibodies were collected at the APS GM/CA Structural Biology facility, at beamline ID-B23 (PC39-17A and PC39-50L), or ID-D23 (PC39-50I and PC39-1 UCA), and three were collected at SSRL 12–2 (PC39-23D, PC39-55C and PC39-50E).

### Structure determination and refinement

Data for most structures were processed using HKL-2000 [105], except for the PC39-1 UCA and PC39-50E datasets, for which XDS [106] was used. Molecular replacement for the structure of PC39-23D was carried out using Phaser [107], with a loop-truncated version of PDB ID: 4NPY, divided into variable and constant regions, as the initial search model. Fab 23D was in turn used as the search model for molecular replacement of the UCA, 17A, 50I, 50L, and 50E. PC39-50E was used as the search model for the molecular replacement for 55C. Model building and refinement were conducted in Coot [108] and PHENIX [109], respectively. Pymol (PyMOL v1.7 Enhanced for Mac OS X) was used for the rendering of images, and the structures were validated with MolProbity [110].

### Envelope gene cloning and mutagenesis

Libraries of full-length envelope genes were isolated by reverse transcription-PCR (RT-PCR) from cryopreserved longitudinal plasma samples from donor PC39. The swarm analysis protocol was described previously [111] and is an application of the clonal analysis procedure developed by Monogram Biosciences (South San Francisco, CA). Briefly, the population of viral envelope genes present in the patient plasma was amplified by RT-PCR and then cloned into expression vectors. To test individual clones derived from the envelope population, the DNA was diluted and retransformed in bacteria, and individual clones were selected and screened for infectivity using the Monogram Biosciences co-receptor tropism screening assay. Pseudo-typed viruses containing cloned envelope genes were prepared from each plasma sample in HEK-293 cells. Viruses from individual clones were screened for infectivity and chemokine receptor tropism in U87 cells transfected with CD4 and the CCR5 or CXCR4 chemokine receptors. Ten to 12 envelopes with high infectivity were selected from each individual and evaluated for autologous neutralization by PC39-1 mAbs at Monogram Biosciences in a micro-neutralization assay using single round of replication pseudoviruses. This assay was based on the 96-well pseudotyped HIV-1 neutralization assay and was modified for screening 15 μL of B-cell culture supernatants in a 384-well format. Briefly, pseudoviruses capable of a single round of infection were produced by co-transfection of HEK-293 cells with a subgenomic plasmid, pHIV-1lucΔu3, that incorporates a firefly luciferase indicator gene and a second plasmid, PC0XAS that expressed HIV-1 Env clones. Following transfection, pseudoviruses were harvested and used to infect U87 cell lines expressing CD4 plus the CCR5 and CXCR4 coreceptors. Virus infectivity was determined 72h after inoculation by measuring amount of luciferase activity. Full-length autologous *env* genes were synthesized for representative clones of each time point using GeneArt gene synthesis services (Life Technologies), then cloned into pcDNA3.1 vector (Life Technologies) for pseudovirus production. Mutagenesis was performed using Quikchange site-directed mutagenesis kit (Agilent Technologies). Individual full-length envelope gene sequences are available from GenBank database (accession numbers: OQ747368—OQ747574).

### Full-length *env* amplification, library preparation, and sequencing

HIV-1 envelope genes were amplified and sequenced as described [112]. Plasma was layered onto 200 μL of 20 per cent sterile-filtered sucrose in 2-mL screw-cap microcentrifuge tubes. Virions were pelleted through the sucrose cushion at 23,500 g for 1 hour at 4°C, and the supernatant discarded. On ice, 140 μL sterile phosphate-buffered saline (pH = 7.4) was applied to the remaining viral pellet and allowed to stand for 1 hour. The loosened viral pellets were then resuspended in this volume and used as input into the QIAamp Viral RNA Mini Kit (Qiagen), which was used to extract total HIV-1 RNA according to the manufacturer’s instructions. Viral RNA was eluted from the QIAmp columns in 55 μL AVE buffer. An 8 μL sub-aliquot of the eluted viral RNA was added directly to cDNA synthesis reactions to avoid any degradation from freeze/thaw cycles. Remaining viral RNA was aliquoted and stored at -80°C for later use. cDNA was generated using the SuperScript III First Strand Synthesis System for RT-PCR (Thermo Fisher) using the provided oligo (dT) to prime first-strand synthesis and according to the manufacturer’s protocol. Aliquots of cDNA were stored at -20°C.

HIV-1 *env* amplification was performed using PCR with HPLC-purified primers: Env-F: GAGCAGAAGACAGTGGCAATGA (corresponding to positions 6,207–6,228 in HXB2); and Env-R: CCACTTGCCACCCATBTTATAGCA (corresponding to positions 8,788–8,811 in HXB2). The primers (IDT) were diluted to 20 pmol in 0.1× TE buffer before use. Each reaction consisted of 2 μL HIV-1 cDNA and 48 μL of Advantage 2 PCR reaction mixture (Clontech). The reaction mixture comprised 5 μL 10× SA PCR Buffer, containing 2 mM magnesium acetate, 1 μL of 10 mM dNTP mix, 1 μL each of Env-F and Env-R (at 20 pmol), 39 μL of nuclease free water, and 1 μL of Advantage 2 Polymerase Mix. Reactions were heated to 95°C for 1 minute and then subjected to 35 cycles of PCR using the following parameters: 15-sec denaturation at 95°C, followed by 30-sec annealing at 64°C, followed by 3-min extension at 68°C. After the 35th cycle, the reactions were incubated for 10 min at 68°C and then held at 4°C.

HIV-1 *env* amplicons were purified from PCR reactions using the QIAquick PCR Purification Kit (Qiagen) as described by the manufacturer, and eluted in 30 μL EB buffer (10 mM Tris, pH 8). Replicate PCR reactions for each sample were visualized and quantitated using the 2100 Bioanalyzer System with the DNA 12000 kit (Agilent Technologies), and pooled by sample, until a final mass of >250 ng HIV-1 *env* amplicon was achieved. To remove any residual PCR reagents and primer dimers, the 250 ng of sample was then purified with a 1× volume of AMPure PB beads (Pacific Biosciences) as described by the manufacturer.

SMRTbell template libraries of ∼2.6-kb insert size were prepared according to the manufacturer’s instructions using the SMRTbell Template Prep Kit 1.0 (Pacific Biosciences). A total of 250 ng of AMPure PB bead-purified HIV-1 *env* amplicon was added directly into the DNA damage repair step of the 10-kb Template Preparation and Sequencing (with low-input DNA) protocol. Sequencing primer annealing was performed using the recommended 20:1 primer:template ratio, whereas P5 polymerase binding was performed at a modified polymerase:template ratio of 3:1. HIV-1 *env* SMRTbell libraries were immobilized onto SMRT cells at a starting concentration of 10 pM on chip, loading titrations were performed to achieve optimal sequencing conditions for particular samples as necessary. SMRT sequencing was performed on the PacBio RS II using the C3 sequencing kit with magnetic bead loading and 180-minute movies.

### Full-length *env* sequence analysis

CCS sequences were constructed using the PacBio SMRTportal software (version 2.3), which employs the Quiver algorithm, and .fastq files were used for downstream analysis. The Full-Length Envelope Analysis (FLEA) pipeline [112] was used to error correct these CCS reads, and cluster them into near-identical clusters, inferring High Quality Consensus Sequences (HQCSs) for each cluster. Envelope phylogenies, as well as the dynamics of amino acid frequency evolution, were inferred from these HQCSs. MAFFT v7.164b [96] with manual curation, was used to create a multiple sequence alignment. Gappy regions were manually removed when reconstructing phylogenies, since their alignment is uncertain. Phylogenies were reconstructed with FastTree v2.1 [97] and visualized with FigTree (http://tree.bio.ed.ac.uk/software/figtree/). Frequency kinetic plots and similar analyses were created with custom Mathematica scripts. Raw fastq files are available on the SRA database (BioProject PRJNA943240).

### Production of monomeric and trimeric recombinant Envelope glycoproteins

All Env-derived gp120 proteins were expressed using the HEK-293-F cells (ThermoFisher) according to manufacturer’s instructions. In brief, cell culture supernatants of 293-transfected cells were harvested 4–6 days post-transfection, cleared, filtered and two protease inhibitor tablets (Roche) per liter of supernatant were added to limit proteolysis. Secreted Env Proteins were purified using affinity columns. Gp120 proteins were purified from HEK-293-F supernatants on Galanthus nivalis lectin-bound agarose columns (Vector Laboratories) while PC39 Env trimers were purified over a F105 column as previously described [113]. The eluted glycoproteins were subjected to size exclusion chromatography on HiLoad 16/600 Superdex 200 SEC column (GE Healthcare). Fractions containing the relevant monomers or trimers were concentrated with Amicon Ultra 30,000 MWCO centrifugal filter devices (Millipore, Bedford, MA). Finally, the purified proteins were subjected to sodium dodecyl sulfate-polyacrylamide gel electrophoresis to verify protein purity. PC39 Env NFL constructs PC39 Env NFL constructs (Guenaga) were co-transfected with furin protease into 1L of HEK-293-F cells at 1.2 x 10^6^ cells/mL.

### Quantification and statistical analysis

For all mAb/serum pseudovirus neutralization assays, the IC_50_, or concentration of mAb/dilution of serum needed to obtain 50% neutralization against a given pseudovirus, was calculated using linear regression of the linear part of the neutralization curve. EC_50_ values were calculated using Prism6 (GraphPad). Escape mapping plots were generated and significance testing for resistance properties conducted in Prism6 (GraphPad).

## Supporting information

S1 Fig PC39-1 bnAb isolation and 37-virus panel neutralization results. **(a)** Gating strategy used to sort N332-glycan-dependent memory B cells by flow-cytometry. **(b)** 37-virus panel neutralization results. (top) Neutralization IC_50_ for PC39 Abs for each virus, together with serum ID_50_ (right column) for the same viruses, colored as indicated in the key. (bottom) Breadth for each Ab by virus clade, with an aggregate of mAbs compared to the serum activity (right). NN: not neutralized (>100 μg/ml). **(c)** Sensitivity of HIV isolates to PC39-1 bnAbs, with deeper red indicating greater antibody potency, shown on a phylogeny of HIV clades.
(TIF)

S2 Fig PC39-1 mAb sequence alignment. Alignments of HC (top) and LC (bottom) of the PC39-1 mAbs, relative to the UCA/iGL sequences, with regions indicated and colored by residue, according to the given key, showing accumulated mutations in the mAbs over time. mAb names are colored by antibody CDRH1 insertion and ordered by isolation time point.
(TIF)

S3 Fig 109-virus panel neutralization results. (top) Neutralization IC_50_ for PC39-1 Abs for each virus, together with serum ID_50_ (right column) for the same viruses, colored as indicated in the key. Differences at N301-glycan, N332-glycan, and residues of the _324_GDIR_327_ that are potentially responsible for decreased or absent neutralization by PC39-1 lineage are indicated on the right. NN: not neutralized (>100 μg/ml). NT: not tested. (bottom). Breadth for each Ab by virus clade (summarized from the 109-virus panel), with that of a theoretical combination of PC39-1 bnAbs compared to the serum activity (right).
(TIF)

S4 Fig Alanine scanning mutagenesis of gp120 binding to select PC39-1 mAbs. **(a)** Alanine scanning mutagenesis results for the indicated residues in JR-CSF gp120 against select PC39-1 mAbs, with the fold change in IC_50_ shown, colored by the magnitude of the fold change as shown in the key. NT: not tested. **(b)** PC39-1 mAb binding EC_50_ to the indicated gp120 monomers, colored as indicated.
(TIF)

S5 Fig PC39-1 mAb NGS. **(a)** Phylogeny of PC39-1 HC Ab next generation sequencing (NGS) from donor PC39, colored by time point with the mAbs indicated with black labels. **(b)** Phylogeny of PC39-1 HC Ab NGS from donor PC39, colored by CDRH1 insertion length with the mAbs indicated with black dots and labels. **(c)** Box and whisker plot of percentage HC (V_H_+J_H_) nucleotide mutations (versus the unmutated common ancestor) in the PC39-1 lineage over time. **(d)** PC39-1 bnAb lineage emergence and evolution compared between bulk-NGS (blue) and scRNA-seq (orange) datasets.
(TIF)

S6 Fig Number of peripheral memory B cells profiled by single-cell RNA-sequencing with corresponding density plot showing B cell abundance in donor PC39 at each time-point. The variable number of sampled cells between timepoints could have potentially resulted in the observed different number of PC39-1 lineage members identified between timepoints.
(TIF)

S7 Fig Identification of Memory B Cell Phenotypes from Gene Expression Data. **(a)** CellTypist annotation of scRNAseq dataset with the “Immune_All_High” model, used for preliminary cell-type identification. **(b)** Predicted cell type from **(a)** for each cluster from low resolution Louvain clustering of the scRNAseq dataset. **(c)** Top left: UMAP projection colored by time-point. Top right: CellTypist annotation of scRNAseq dataset with the “Immune_All_Low” model. Bottom left: CellTypist annotation of scRNAseq dataset with the “COVID19_Immune_Landscape” model. Bottom Right: CellTypist annotation of scRNAseq dataset with the “Healthy_COVID19_PBMC” model. **(d)** UMAP projection of scRNAseq dataset with PC39-1 bnAb lineage members colored in red. **(e)** Frequency of each cell type at each indicated time-point post-infection within PC39-1 lineage members isolated with 10X Genomics.
(TIF)

S8 Fig PC39-1 bnAb lineage members show little auto-/poly-reactivity. **(a)** Binding ELISA curves generated with the indicated antibodies and autoantigens. **(b)** PC39-1 Ab binding EC_50_ values (μg/mL) to the indicated autoantigens. NT: not tested. **(c)** Glycan array binding by PC39-1 UCA mAb shows no specific reactivity to glycans. NCFGv5.5: National Center for Functional Glycomics Version 5.5.
(PDF)

S9 Fig PC39 virus sequencing. **(a)** *env* phylogeny from PC39, colored by mpi as indicated, and estimated by maximum likelihood from full-length *env* PacBio high-quality consensus sequences (HQCSs) (bubble size represents sample proportion). Sanger sequences (Monogram Biosciences Lab Corp) are indicated by dashed lines, which are represented according to how well they are neutralized, as indicated. **(b)** *env* phylogeny from the source partner, with the two founder variants from PC39 indicated in red according to their phylogenetic relationship to the viral population within the source. **(c)** (top) Amino-acid residues at each position in Env compared to Founder 1 are presented as stacked frequency bar graphs for each time point. Identical amino acids are marked grey and mutations are color-coded as indicated. Founder 2 is clearly visible at 4MPI. (bottom) Potential N-linked glycosylation site (PNGS) locations in Env sequences from PC39 are presented as stacked frequency bar graphs for each time point.
(TIF)

S10 Fig PC39-1 UCA and pre-insertion Ab binding to autologous gp120s. **(a)** BLI curves generated with the indicated antibodies immobilized on anti-human IgG Fc sensors and the indicated gp120s. **(b)** Binding ELISA curves generated with the indicated antibodies and gp120s. **(c)** Neutralization IC_50_ for PC39-1 Abs for each autologous virus. Heterologous IAVIc22 and 92BR020 viruses included as positive control. mAb F105 included as negative control. **(d)** BLI curves generated with the indicated antibodies immobilized on anti-human IgG Fc sensors and the indicated gp120s or NFL constructs.
(TIF)

S11 Fig Mutational scanning of autologous virus. Each of (a-d) shows the effect of mutations in a different viral backbone (replicates shown in different columns). The top panel shows fold increase/decrease while the bottom shows IC_50_ (μg/ml). Grey shading indicates wild-type virus, red indicates a decrease in neutralization, and green an increase in sensitivity. Each point represents a measurement against a PC39-1 Ab, stars are control Ab (green: PGT121, blue: VRC01, orange: PGT128, red: PGT135, purple: PGV04).
(TIF)

S12 Fig Comparison of V3-glycan targeting antibody structures. **(a)** Ribbon representation of N332-targeting antibodies, with LCs in grey, HCs in lavender, CDRH1 in yellow, CDRH2 in orange, and CDRH3 in red. PC39-1 bnAb structures (bottom) are shown for comparison. **(b)** Cartoon representation of PC39-1 lineage bnAbs 50E (blue), 50L (purple), and 23D (orange) with ball-and-stick representations of Trp residues on CDRH1 and CDRH3. Closest distances between the Trp indoles are shown in Å.
(TIF)

S13 Fig Antibody insertion statistics compiled from a large-scale repertoire sequencing study [90] and previously studied Protocol C donors [21, 23, 27]. **(a)** Frequency of antibody sequences containing an insertion in the IgG repertoire of 10 healthy donors, each dot represents a separate replicate. **(b)** Frequency of antibody sequences containing an insertion in the IgG repertoire of 10 healthy donors for each indicated VH gene family, colored by donor, each dot represents a separate replicate. **(c)** Frequency of antibody sequences containing an insertion in the IgG repertoire of 10 healthy donors for each indicated VH gene. **(d)** Frequency of each indicated insertion length (amino acid) for antibody sequences containing an insertion within the IgG repertoire of 10 healthy donors for each indicated antibody VH gene. **(e)** In-frame insertion frequencies in the IgG repertoire of 10 healthy donors from U.S.A., healthy donors from Sub-Saharan Africa and HIV-infected Protocol C bnAb donors. **(f)** In-frame insertion frequencies in the peripheral IgG repertoire of HIV-infected Protocol C bnAb donors over-time.
(TIF)

S1 Table X-ray data collection and refinement statistics for PC39-17A and PC39-50I Fab structures. (TIF)

S2 Table X-ray data collection and refinement statistics for PC39-50E and PC39-50L Fab structures. (TIF)

S3 Table X-ray data collection and refinement statistics for PC39-55C and PC39-1 UCA Fab structures. (TIF)

S4 Table X-ray data collection and refinement statistics for PC39-23D Fab structure. (TIF)
