# Supplementary figures and images for: Antigen pressure from two founder viruses induces multiple insertions at a single antibody position to generate broadly neutralizing HIV antibodies
Source: PLoS Pathog. 2023 Jun 29;19(6):e1011416. doi: 10.1371/journal.ppat.1011416 (PMC10309625; doi:10.1371/journal.ppat.1011416)

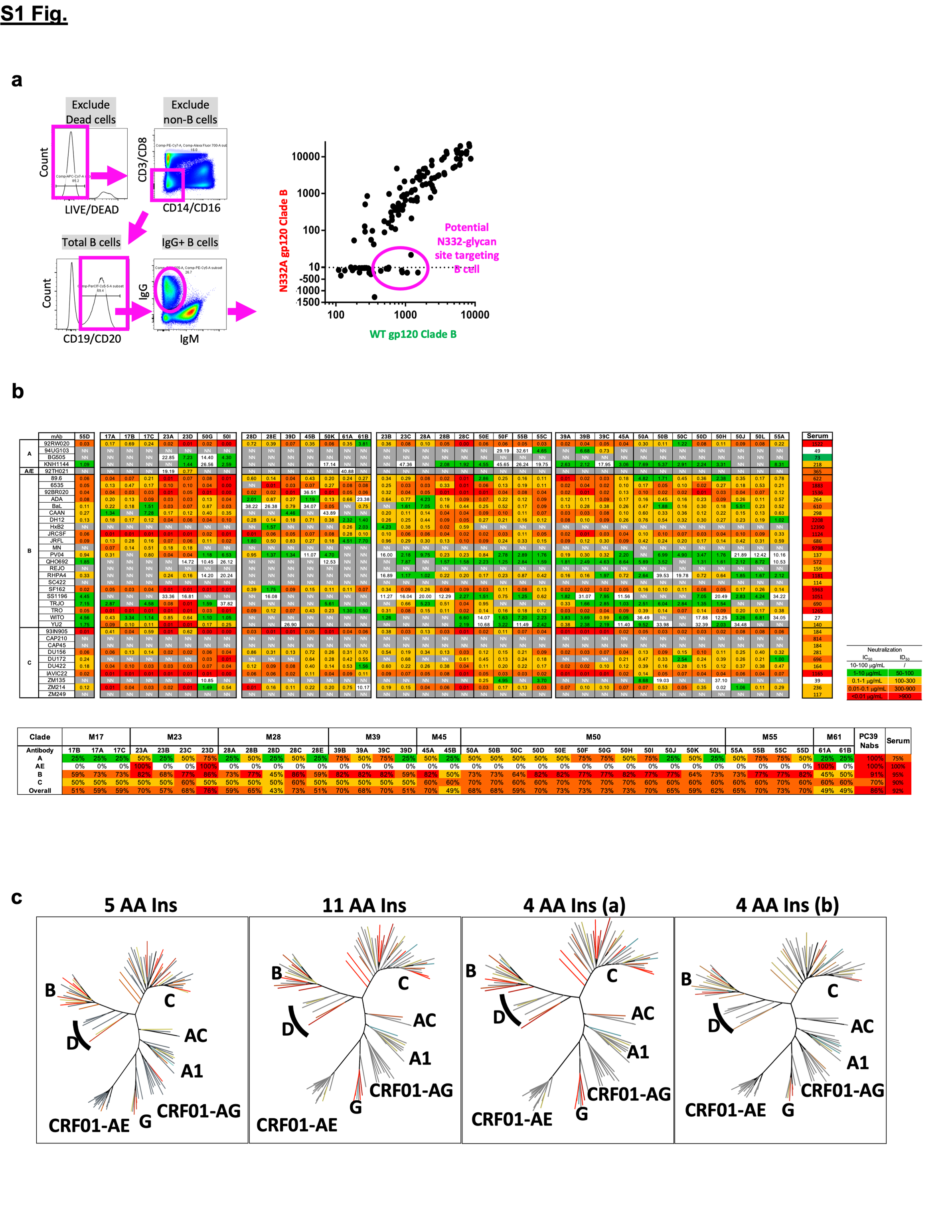

Supplement: S1 Fig — (a) Gating strategy used to sort N332-glycan-dependent memory B cells by flow-cytometry. (b) 37-virus panel neutralization results. (top) Neutralization IC50 for PC39 Abs for each virus, together with serum ID50 (right column) for the same viruses, colored as indicated in the key. (bottom) Breadth for each Ab by virus clade, with an aggregate of mAbs compared to the serum activity (right). NN: not neutralized (>100 μg/ml). (c) Sensitivity of HIV isolates to PC39-1 bnAbs, with deeper red indicating greater antibody potency, shown on a phylogeny of HIV clades. (TIF) [file ppat.1011416.s001.tif]

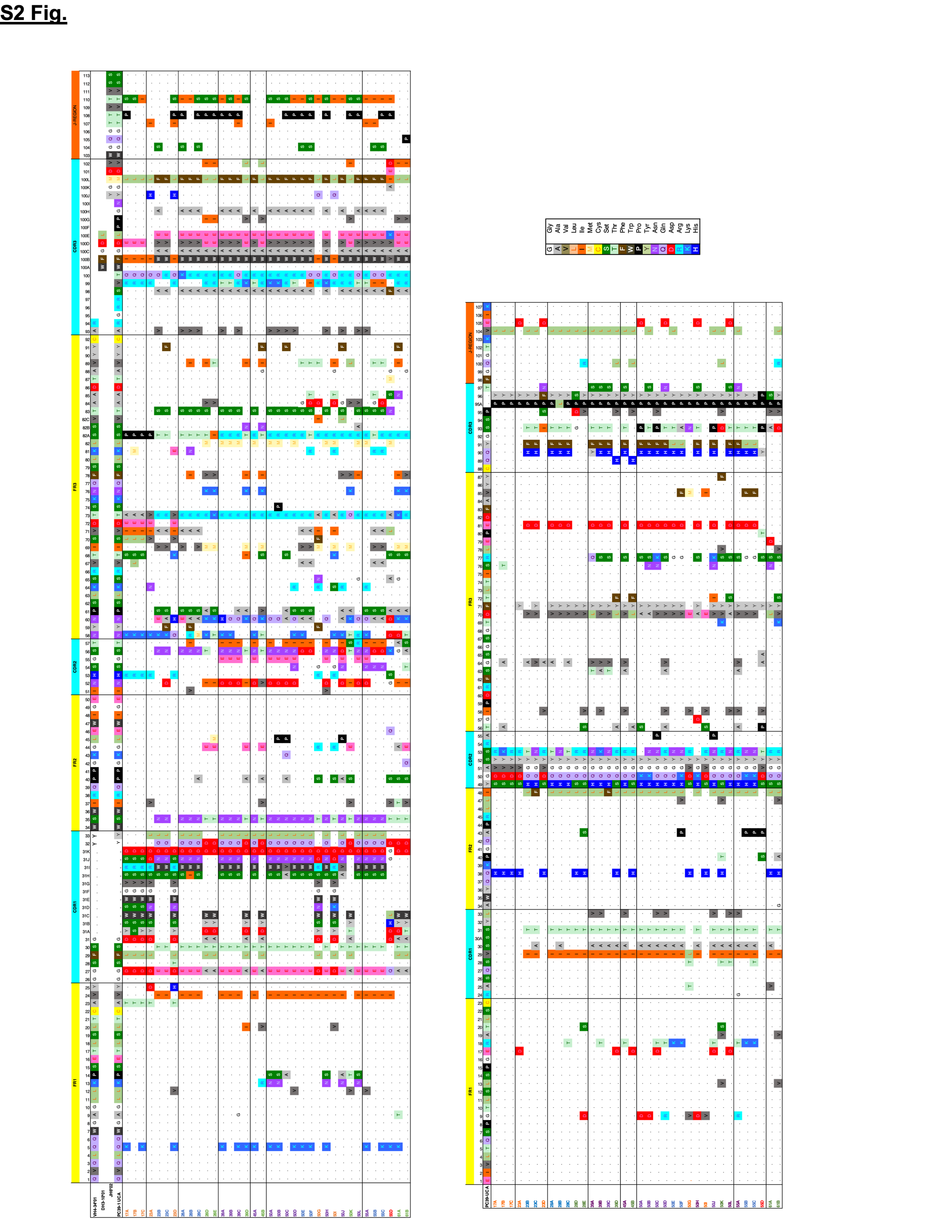

Supplement: S2 Fig — Alignments of HC (top) and LC (bottom) of the PC39-1 mAbs, relative to the UCA/iGL sequences, with regions indicated and colored by residue, according to the given key, showing accumulated mutations in the mAbs over time. mAb names are colored by antibody CDRH1 insertion and ordered by isolation time point. (TIF) [file ppat.1011416.s002.tif]

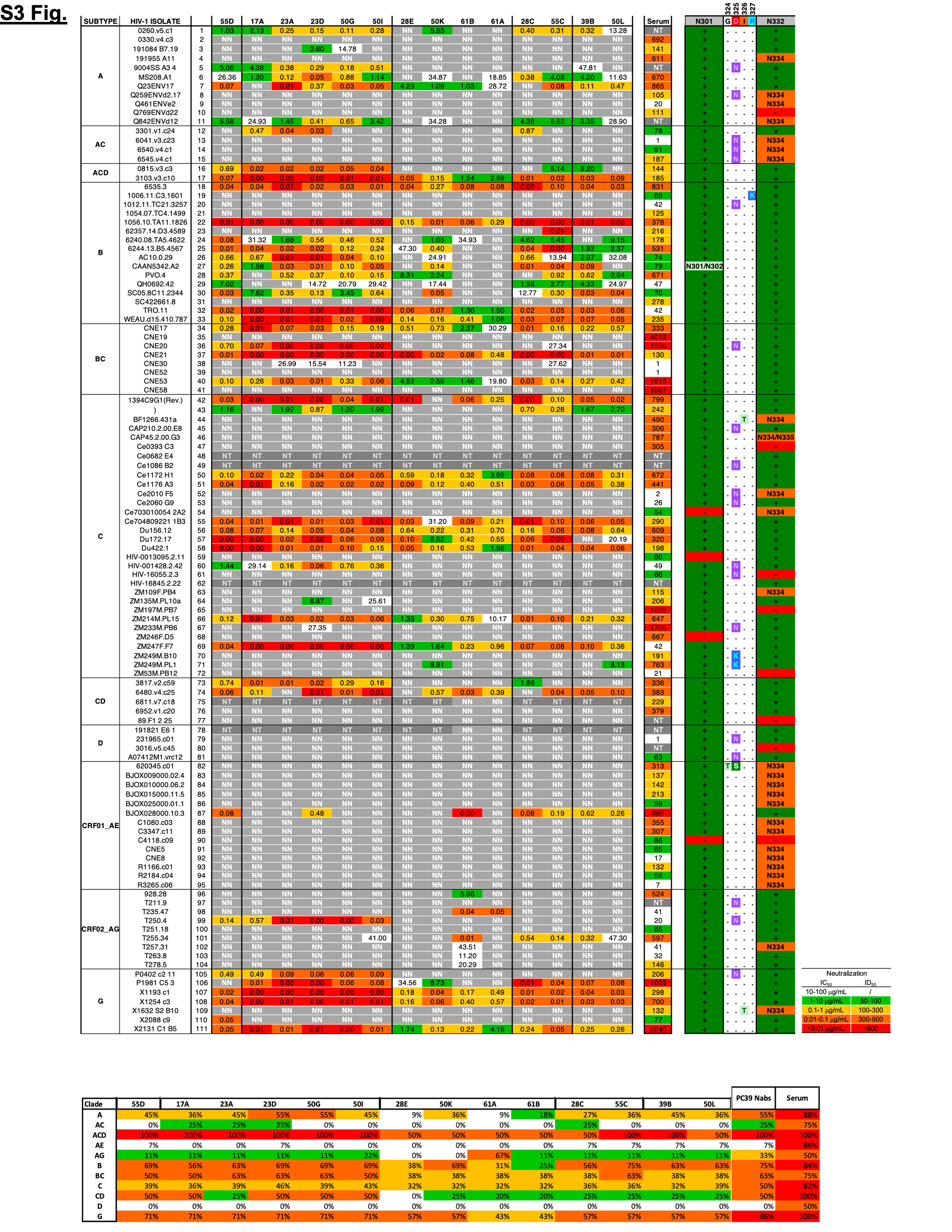

Supplement: S3 Fig — (top) Neutralization IC50 for PC39-1 Abs for each virus, together with serum ID50 (right column) for the same viruses, colored as indicated in the key. Differences at N301-glycan, N332-glycan, and residues of the 324GDIR327 that are potentially responsible for decreased or absent neutralization by PC39-1 lineage are indicated on the right. NN: not neutralized (>100 μg/ml). NT: not tested. (bottom). Breadth for each Ab by virus clade (summarized from the 109-virus panel), with that of a theoretical combination of PC39-1 bnAbs compared to the serum activity (right). (TIF) [file ppat.1011416.s003.tif]

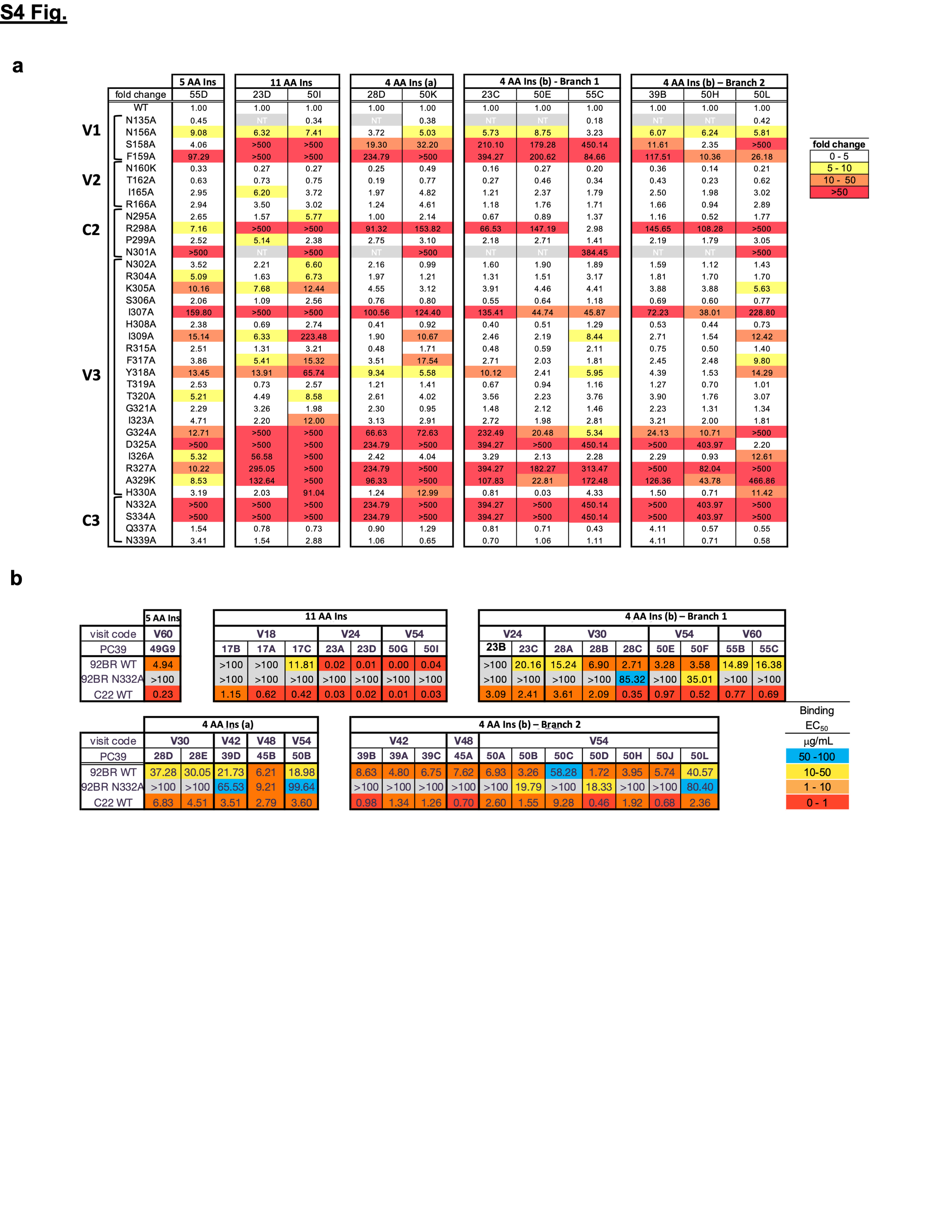

Supplement: S4 Fig — (a) Alanine scanning mutagenesis results for the indicated residues in JR-CSF gp120 against select PC39-1 mAbs, with the fold change in IC50 shown, colored by the magnitude of the fold change as shown in the key. NT: not tested. (b) PC39-1 mAb binding EC50 to the indicated gp120 monomers, colored as indicated. (TIF) [file ppat.1011416.s004.tif]

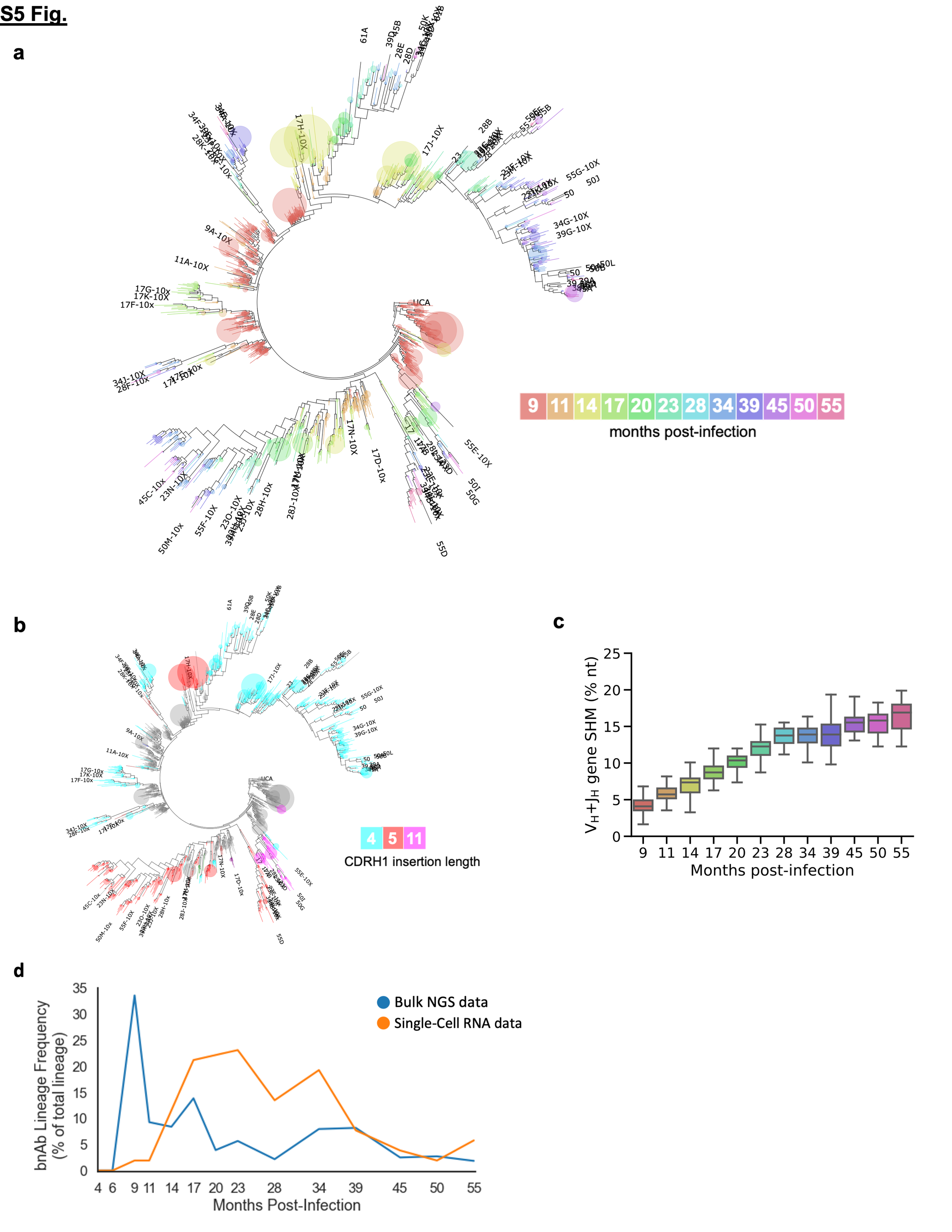

Supplement: S5 Fig — (a) Phylogeny of PC39-1 HC Ab next generation sequencing (NGS) from donor PC39, colored by time point with the mAbs indicated with black labels. (b) Phylogeny of PC39-1 HC Ab NGS from donor PC39, colored by CDRH1 insertion length with the mAbs indicated with black dots and labels. (c) Box and whisker plot of percentage HC (VH+JH) nucleotide mutations (versus the unmutated common ancestor) in the PC39-1 lineage over time. (d) PC39-1 bnAb lineage emergence and evolution compared between bulk-NGS (blue) and scRNA-seq (orange) datasets. (TIF) [file ppat.1011416.s005.tif]

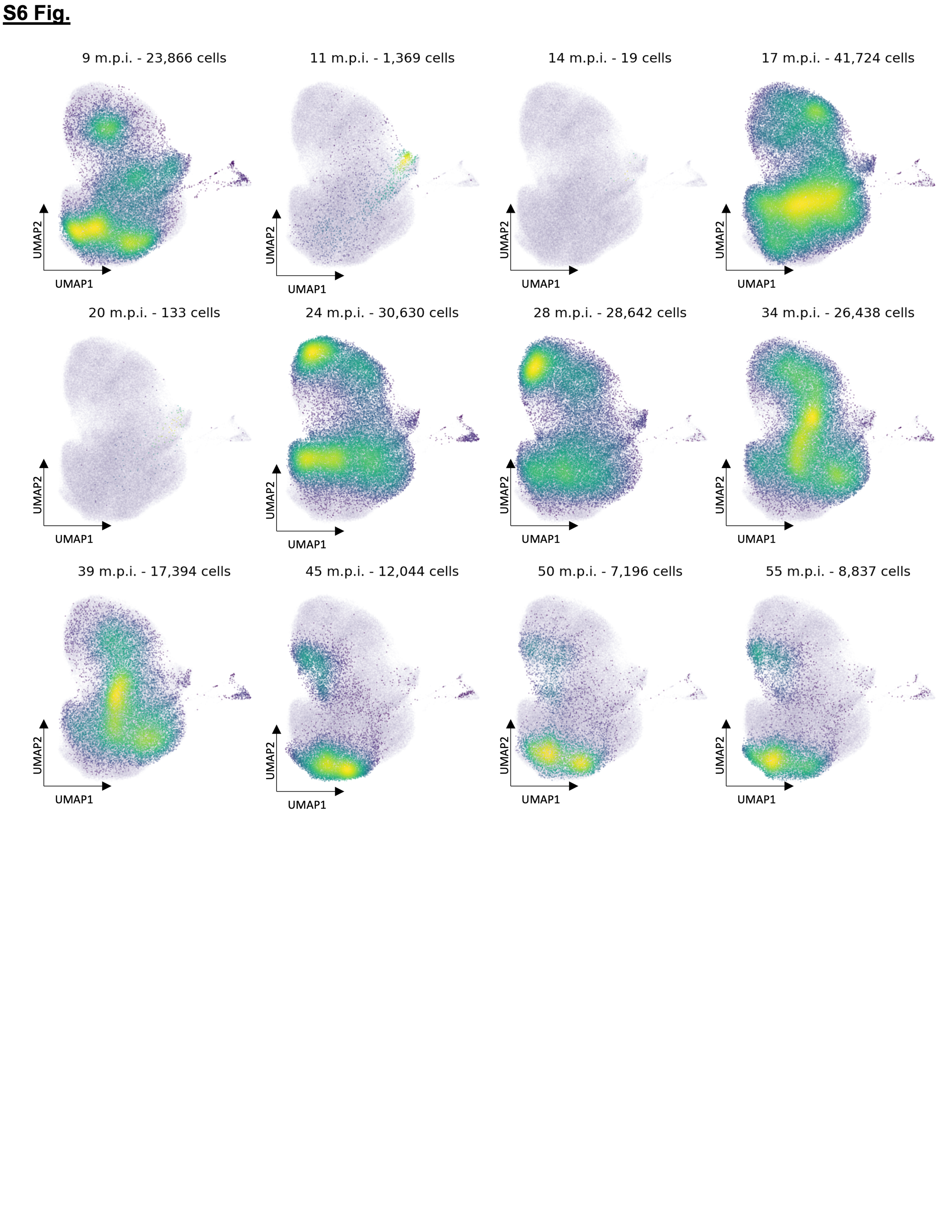

Supplement: S6 Fig — The variable number of sampled cells between timepoints could have potentially resulted in the observed different number of PC39-1 lineage members identified between timepoints. (TIF) [file ppat.1011416.s006.tif]

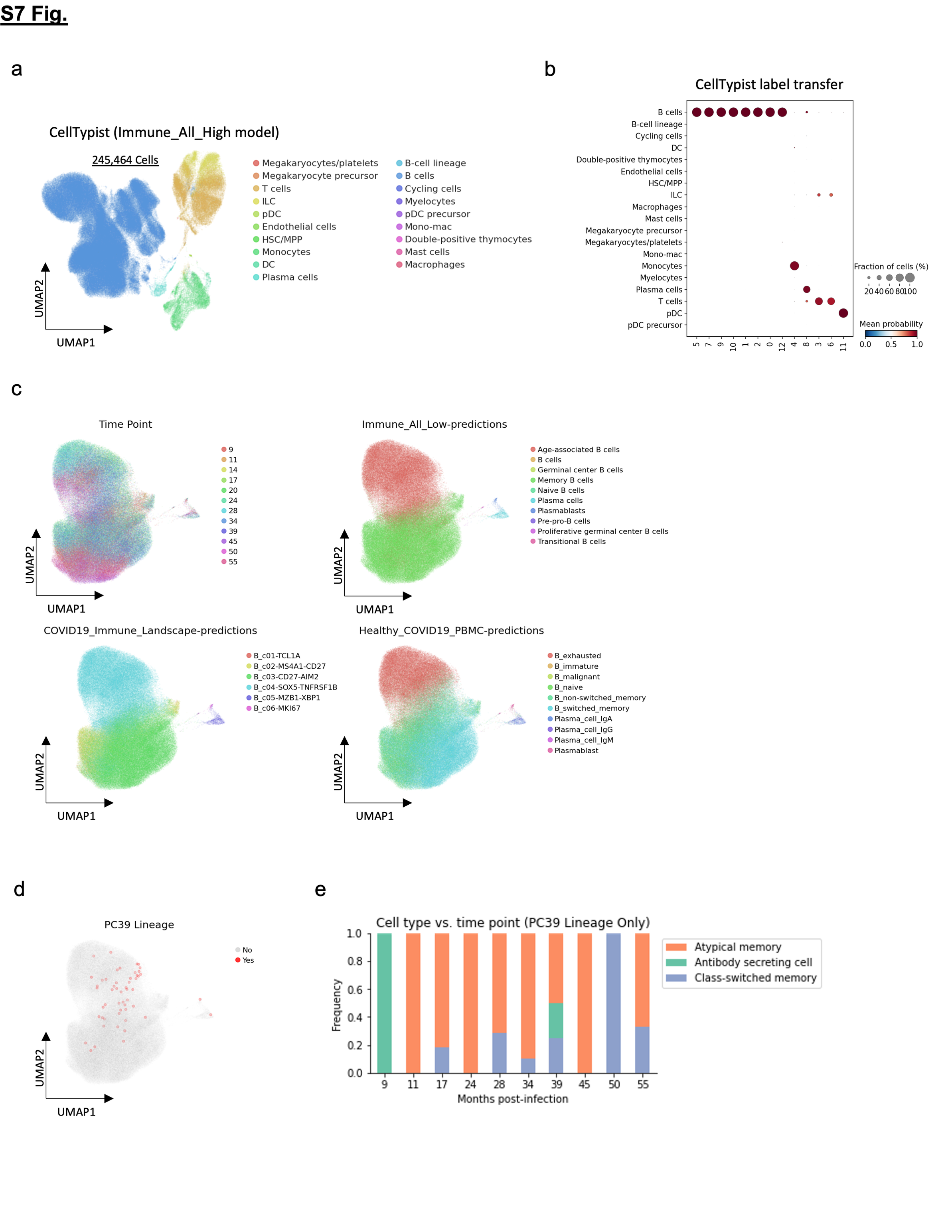

Supplement: S7 Fig — (a) CellTypist annotation of scRNAseq dataset with the “Immune_All_High” model, used for preliminary cell-type identification. (b) Predicted cell type from (a) for each cluster from low resolution Louvain clustering of the scRNAseq dataset. (c) Top left: UMAP projection colored by time-point. Top right: CellTypist annotation of scRNAseq dataset with the “Immune_All_Low” model. Bottom left: CellTypist annotation of scRNAseq dataset with the “COVID19_Immune_Landscape” model. Bottom Right: CellTypist annotation of scRNAseq dataset with the “Healthy_COVID19_PBMC” model. (d) UMAP projection of scRNAseq dataset with PC39-1 bnAb lineage members colored in red. (e) Frequency of each cell type at each indicated time-point post-infection within PC39-1 lineage members isolated with 10X Genomics. (TIF) [file ppat.1011416.s007.tif]

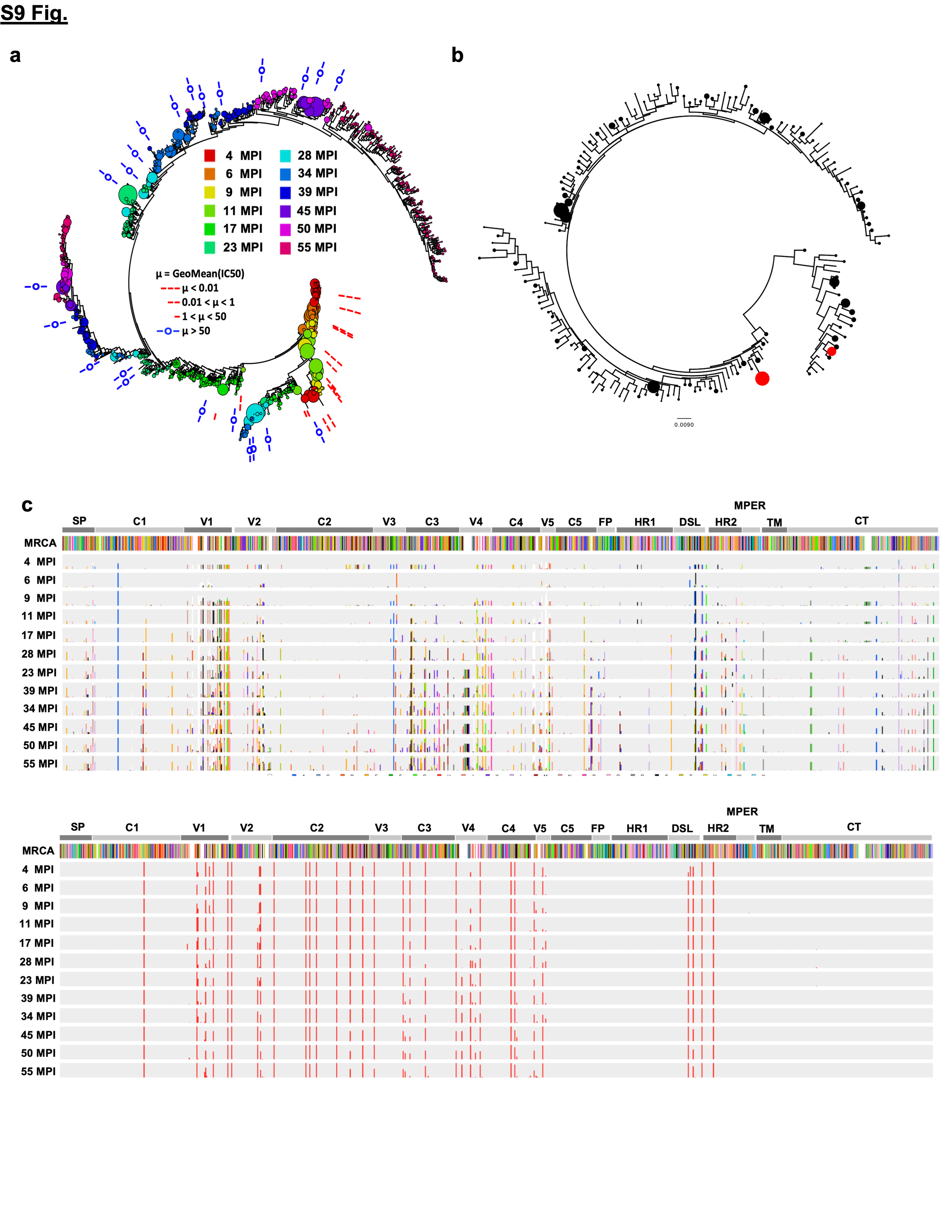

Supplement: S9 Fig — (a) env phylogeny from PC39, colored by mpi as indicated, and estimated by maximum likelihood from full-length env PacBio high-quality consensus sequences (HQCSs) (bubble size represents sample proportion). Sanger sequences (Monogram Biosciences Lab Corp) are indicated by dashed lines, which are represented according to how well they are neutralized, as indicated. (b) env phylogeny from the source partner, with the two founder variants from PC39 indicated in red according to their phylogenetic relationship to the viral population within the source. (c) (top) Amino-acid residues at each position in Env compared to Founder 1 are presented as stacked frequency bar graphs for each time point. Identical amino acids are marked grey and mutations are color-coded as indicated. Founder 2 is clearly visible at 4MPI. (bottom) Potential N-linked glycosylation site (PNGS) locations in Env sequences from PC39 are presented as stacked frequency bar graphs for each time point. (TIF) [file ppat.1011416.s009.tif]

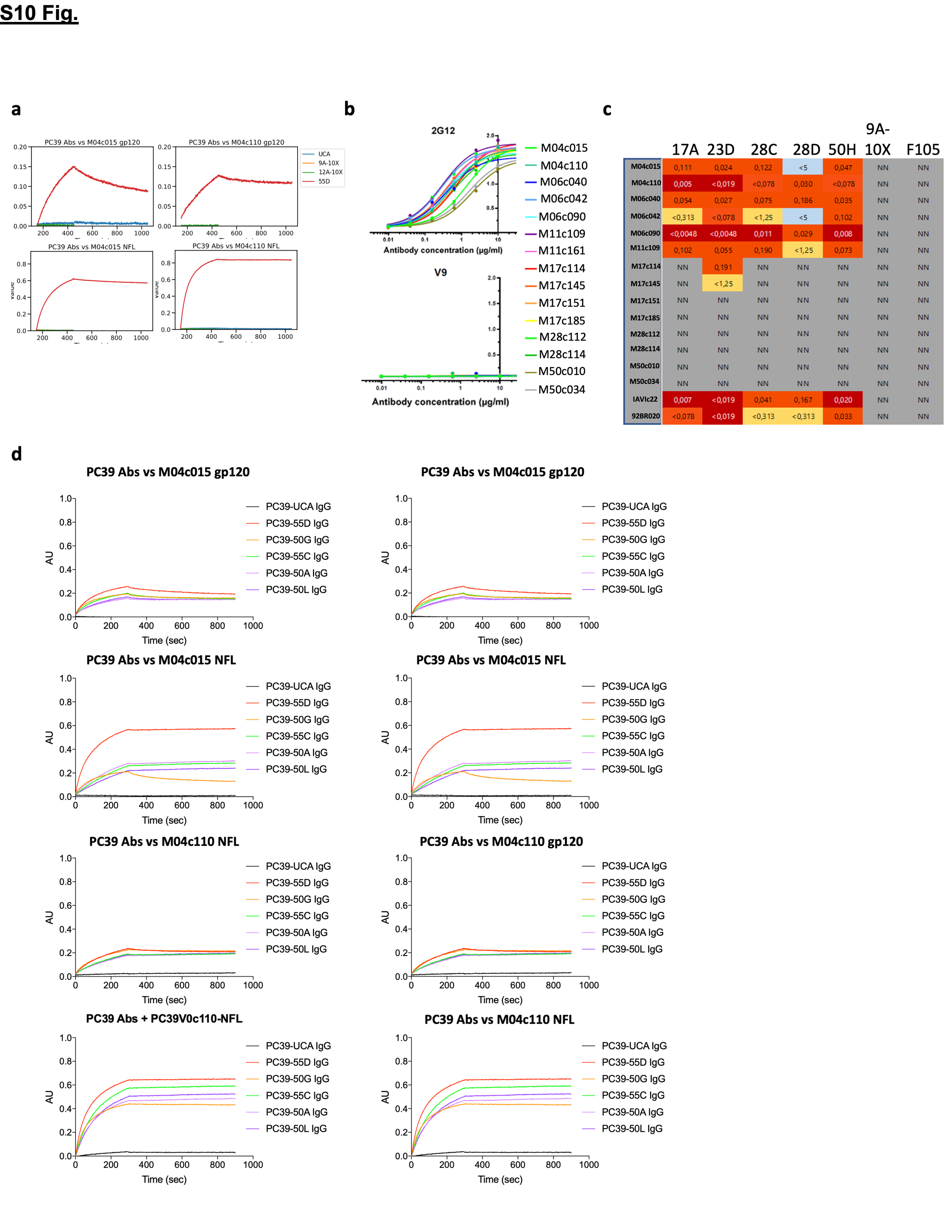

Supplement: S10 Fig — (a) BLI curves generated with the indicated antibodies immobilized on anti-human IgG Fc sensors and the indicated gp120s. (b) Binding ELISA curves generated with the indicated antibodies and gp120s. (c) Neutralization IC50 for PC39-1 Abs for each autologous virus. Heterologous IAVIc22 and 92BR020 viruses included as positive control. mAb F105 included as negative control. (d) BLI curves generated with the indicated antibodies immobilized on anti-human IgG Fc sensors and the indicated gp120s or NFL constructs. (TIF) [file ppat.1011416.s010.tif]

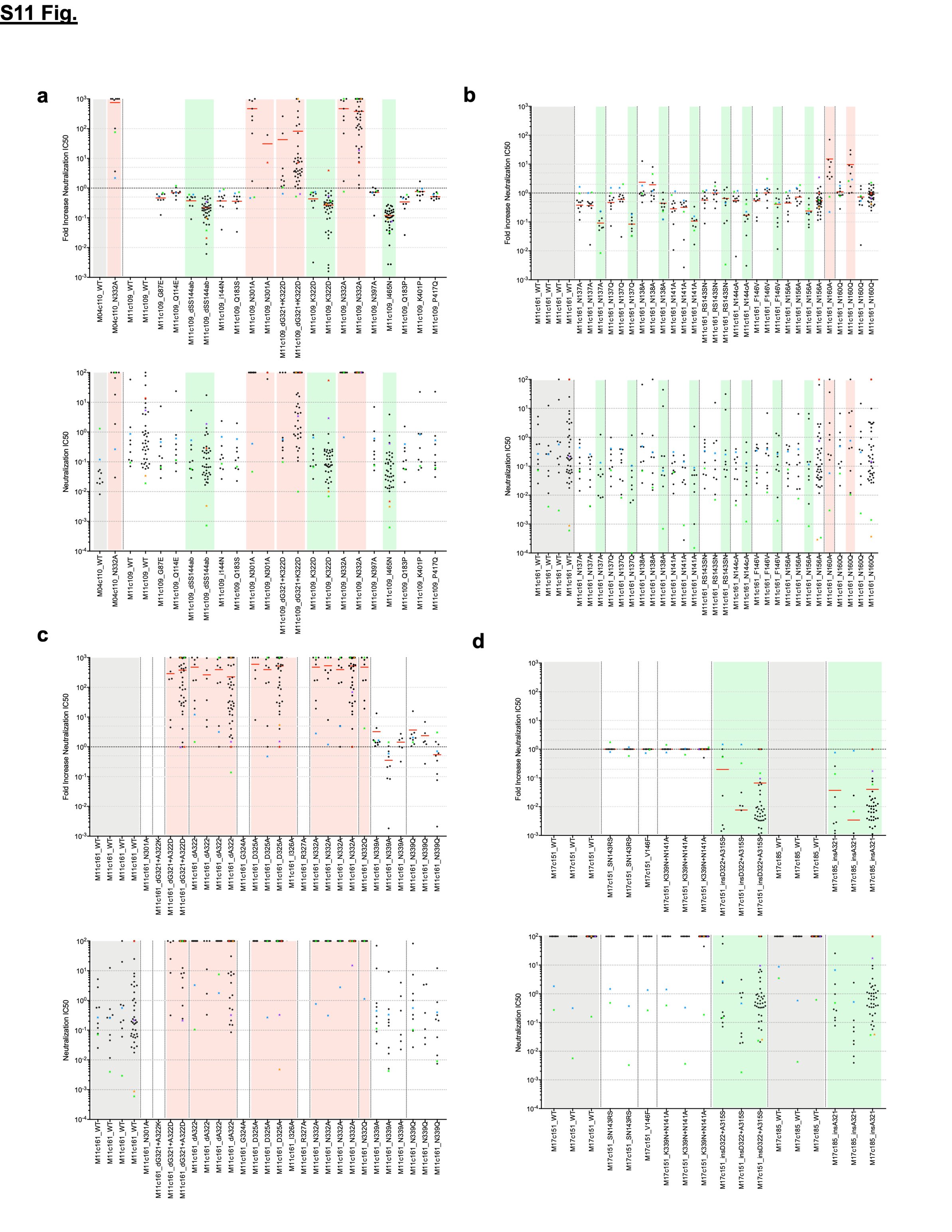

Supplement: S11 Fig — Each of (a-d) shows the effect of mutations in a different viral backbone (replicates shown in different columns). The top panel shows fold increase/decrease while the bottom shows IC50 (μg/ml). Grey shading indicates wild-type virus, red indicates a decrease in neutralization, and green an increase in sensitivity. Each point represents a measurement against a PC39-1 Ab, stars are control Ab (green: PGT121, blue: VRC01, orange: PGT128, red: PGT135, purple: PGV04). (TIF) [file ppat.1011416.s011.tif]

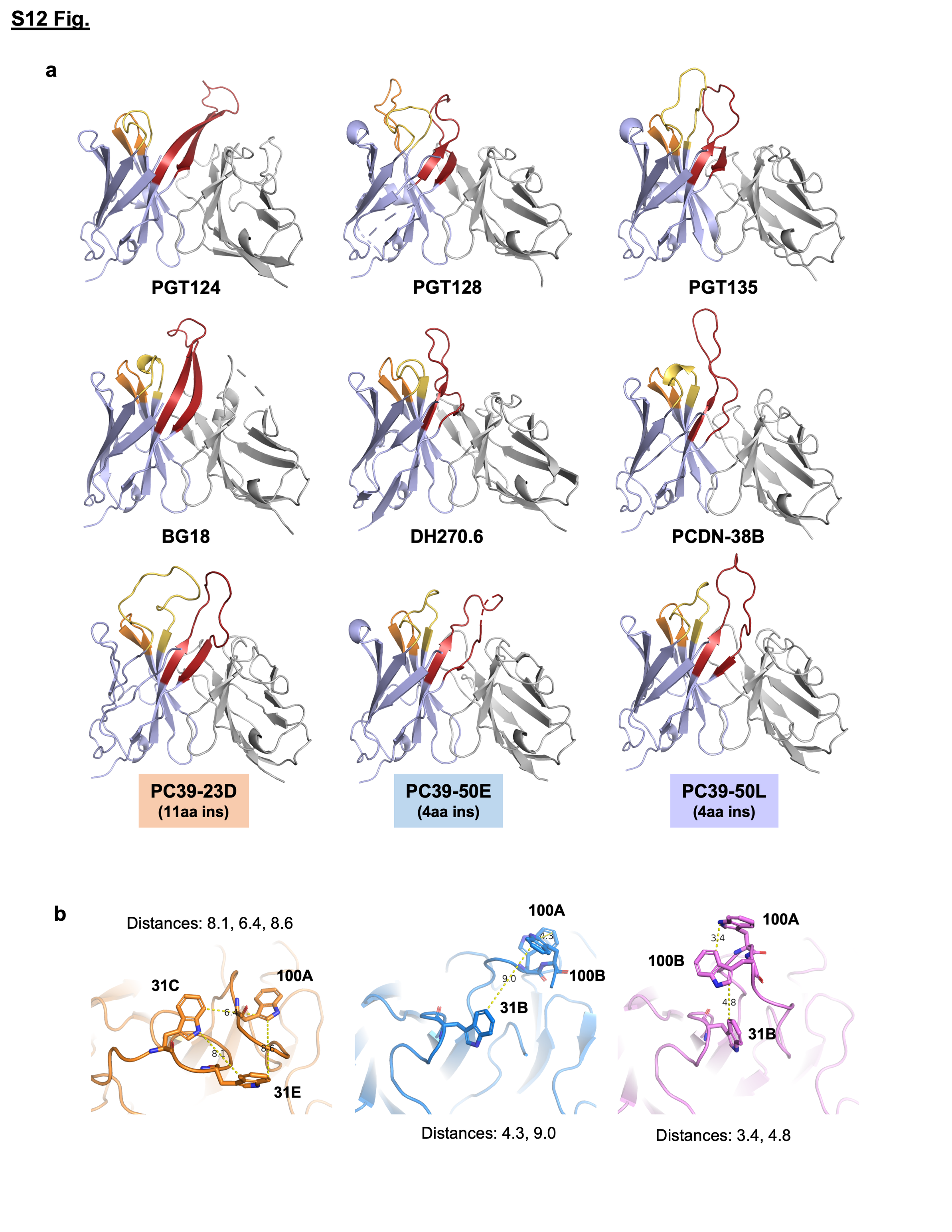

Supplement: S12 Fig — (a) Ribbon representation of N332-targeting antibodies, with LCs in grey, HCs in lavender, CDRH1 in yellow, CDRH2 in orange, and CDRH3 in red. PC39-1 bnAb structures (bottom) are shown for comparison. (b) Cartoon representation of PC39-1 lineage bnAbs 50E (blue), 50L (purple), and 23D (orange) with ball-and-stick representations of Trp residues on CDRH1 and CDRH3. Closest distances between the Trp indoles are shown in Å. (TIF) [file ppat.1011416.s012.tif]

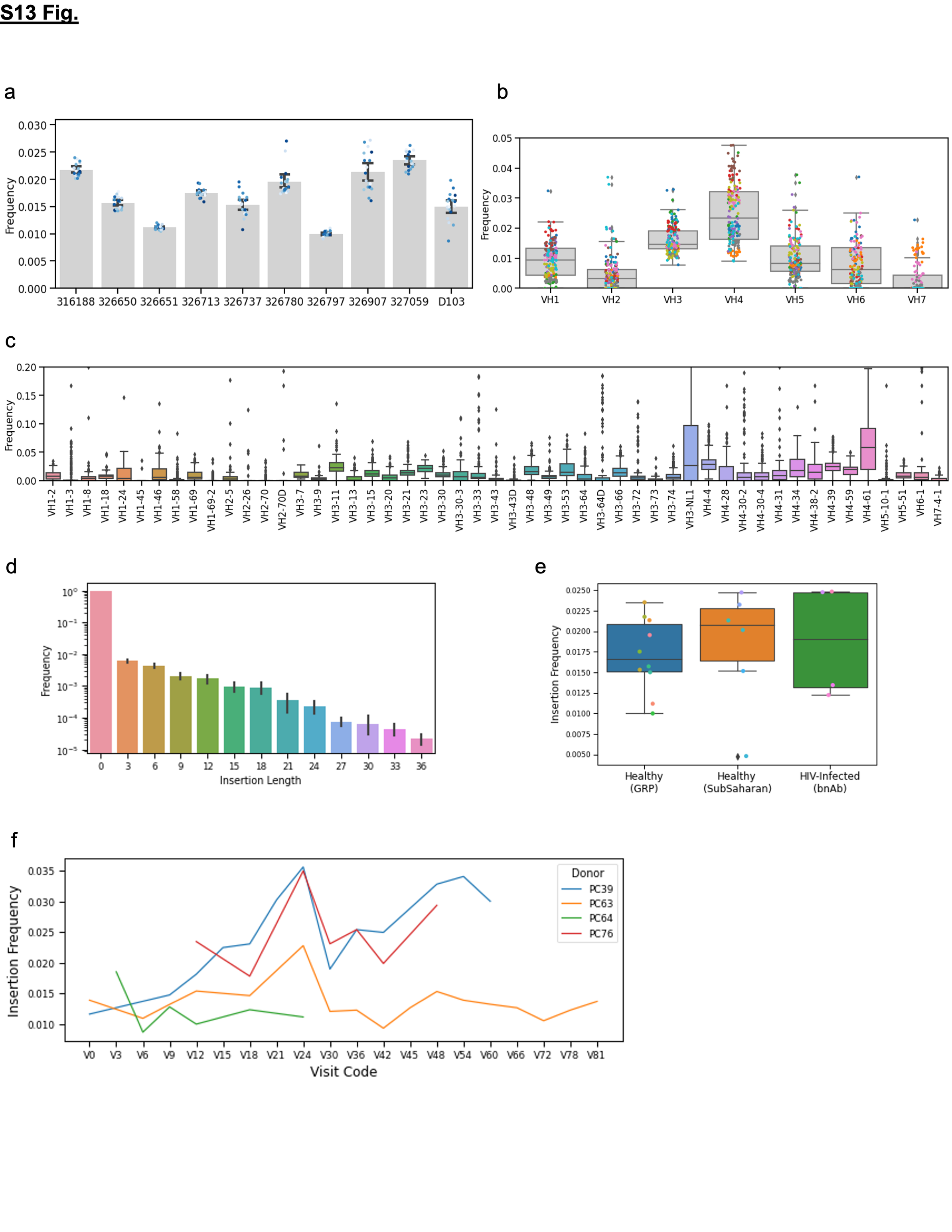

Supplement: S13 Fig — (a) Frequency of antibody sequences containing an insertion in the IgG repertoire of 10 healthy donors, each dot represents a separate replicate. (b) Frequency of antibody sequences containing an insertion in the IgG repertoire of 10 healthy donors for each indicated VH gene family, colored by donor, each dot represents a separate replicate. (c) Frequency of antibody sequences containing an insertion in the IgG repertoire of 10 healthy donors for each indicated VH gene. (d) Frequency of each indicated insertion length (amino acid) for antibody sequences containing an insertion within the IgG repertoire of 10 healthy donors for each indicated antibody VH gene. (e) In-frame insertion frequencies in the IgG repertoire of 10 healthy donors from U.S.A., healthy donors from Sub-Saharan Africa and HIV-infected Protocol C bnAb donors. (f) In-frame insertion frequencies in the peripheral IgG repertoire of HIV-infected Protocol C bnAb donors over-time. (TIF) [file ppat.1011416.s013.tif]

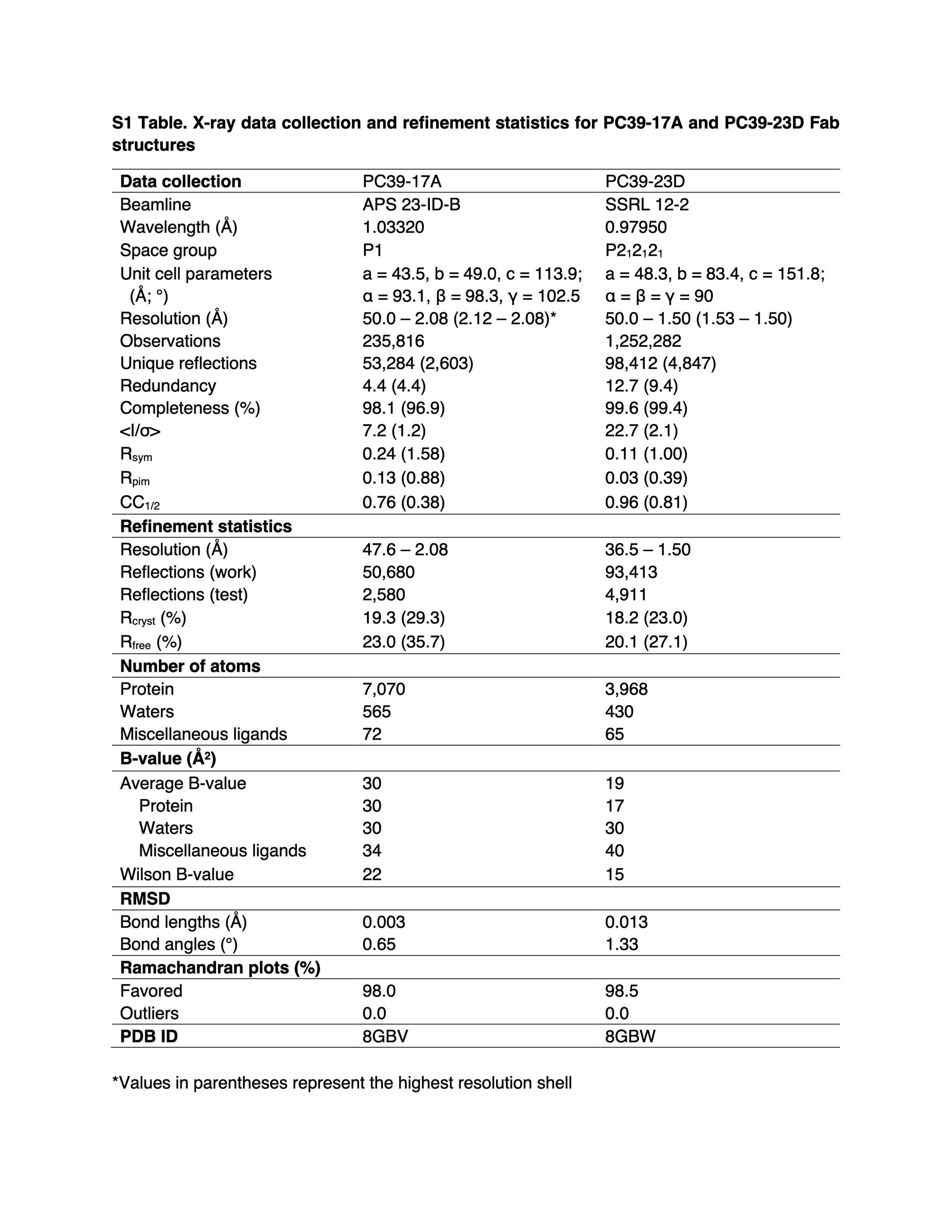

Supplement: S1 Table — (TIF) [file ppat.1011416.s014.tif]

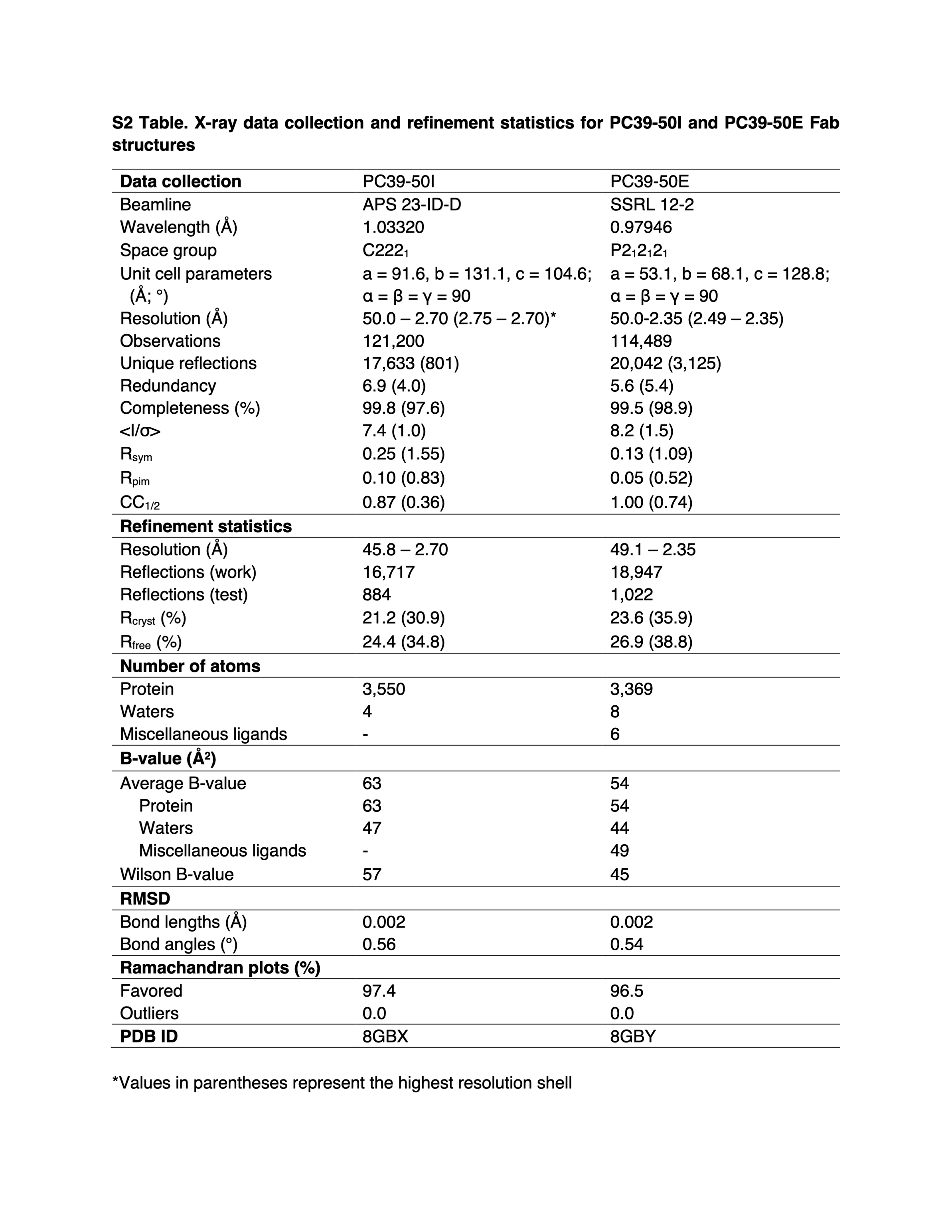

Supplement: S2 Table — (TIF) [file ppat.1011416.s015.tif]

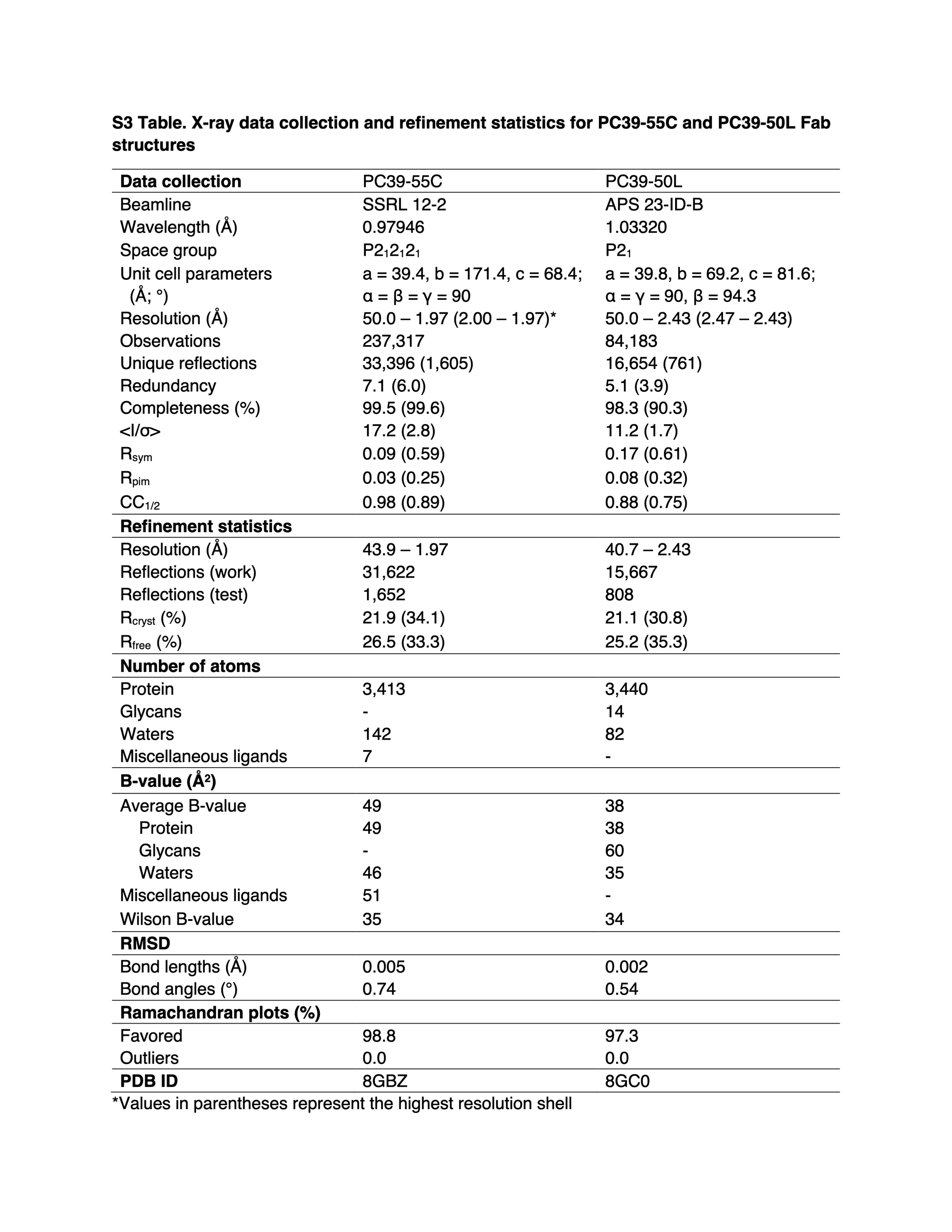

Supplement: S3 Table — (TIF) [file ppat.1011416.s016.tif]

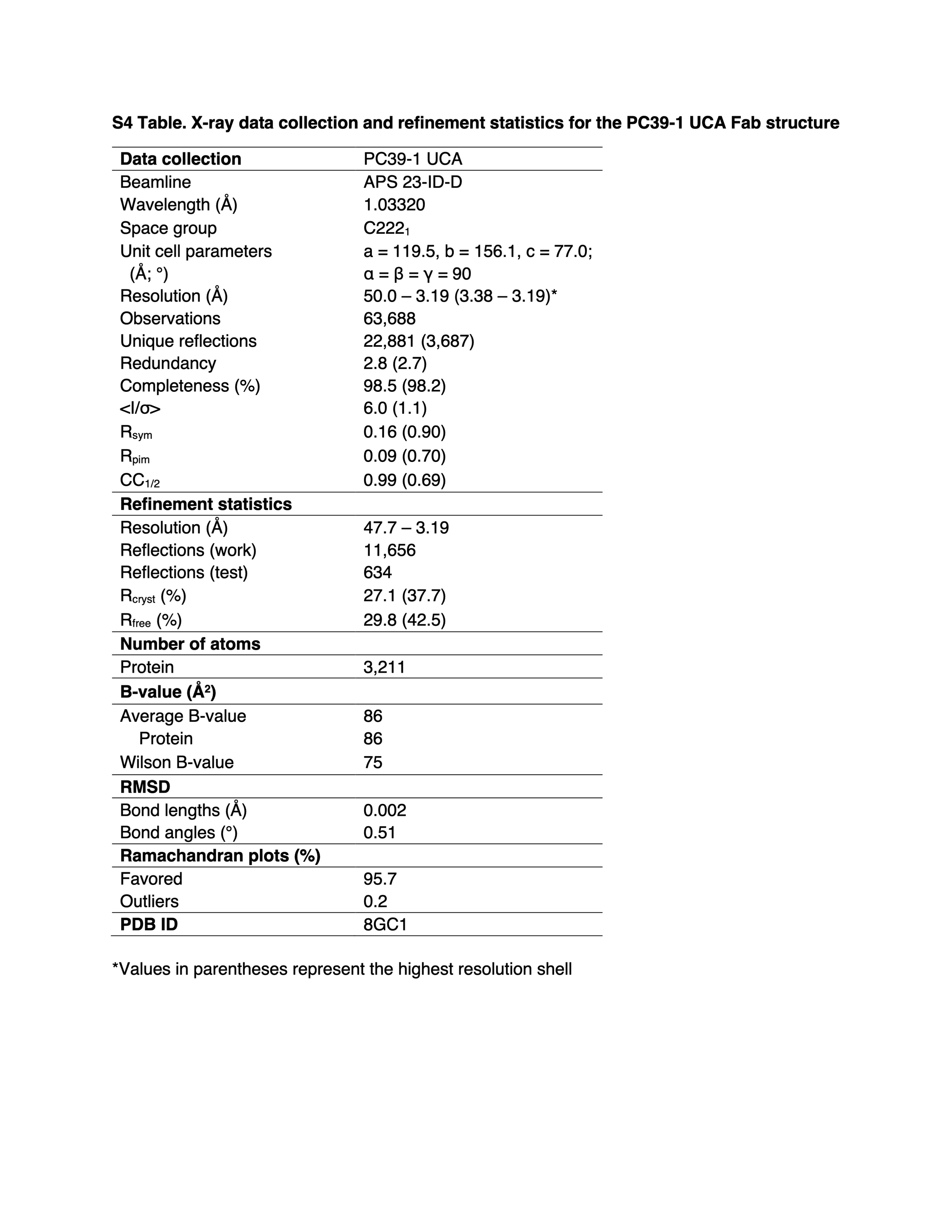

Supplement: S4 Table — (TIF) [file ppat.1011416.s017.tif]
