# Supplementary material for: Antigen pressure from two founder viruses induces multiple insertions at a single antibody position to generate broadly neutralizing HIV antibodies
Source: PLoS Pathog. 2023 Jun 29;19(6):e1011416. doi: 10.1371/journal.ppat.1011416 (PMC10309625; doi:10.1371/journal.ppat.1011416)

S8 Fig.

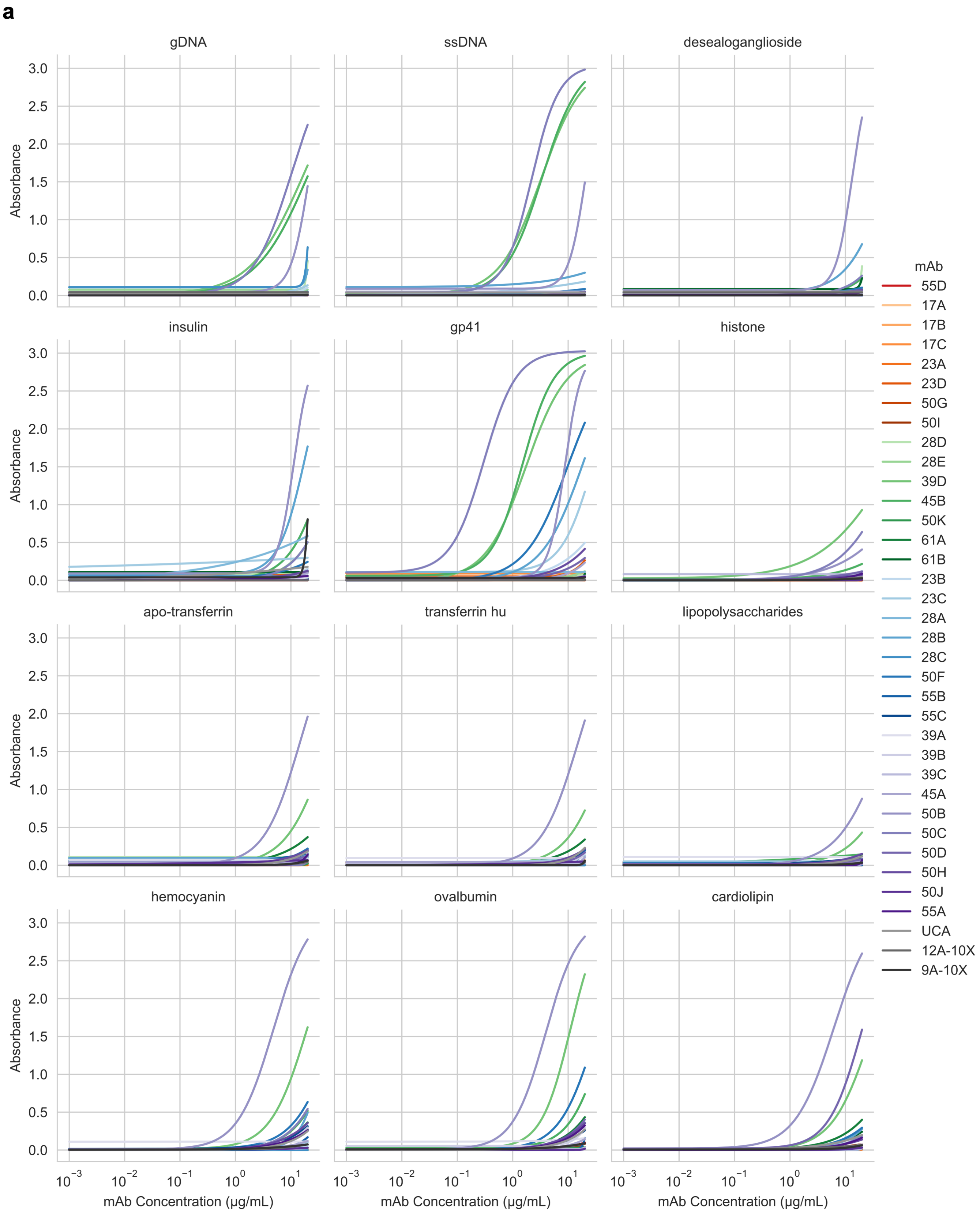
a
gDNA
ssDNA
desealoganglioside
insulin
gp41
histone
apo-transferrin
transferrin hu
lipopolysaccharides
hemocyanin
ovalbumin
cardiolipin
Absorbance
mAb Concentration (µg/mL)
mAb
55D
17A
17B
17C
23A
23D
50G
50I
28D
28E
39D
45B
50K
61A
61B
23B
23C
28A
28B
28C
50F
55B
55C
39A
39B
39C
45A
50B
50C
50D
50H
50J
55A
UCA
12A-10X
9A-10X

S8 Fig. (Continued)

b

Insertion	mAb	LPS	Histone	Cardiolipin	GD_{1a}	Transferin	Apo Trsfrn	OVA	Insulin	KLH	gDNA	ssDNA	gp41
5	55D	>20	>20	>20	>20	>20	>20	>20	>20	>20	>20	>20	>20
	17A	>20	>20	>20	>20	>20	>20	NT	>20	>20	>20	>20	NT
	17B	>20	>20	>20	>20	>20	>20	NT	>20	>20	>20	>20	NT
	17C	>20	>20	>20	>20	>20	>20	NT	>20	>20	>20	>20	>20
11	23A	>20	>20	>20	>20	>20	>20	NT	>20	>20	>20	>20	>20
	23D	>20	>20	>20	>20	>20	>20	NT	>20	>20	>20	>20	>20
	50G	>20	>20	>20	>20	>20	>20	>20	>20	>20	>20	>20	>20
	50I	>20	>20	>20	>20	>20	>20	>20	>20	>20	>20	>20	>20
	28D	>20	>20	>20	>20	>20	>20	NT	>20	>20	>20	>20	>20
	28E	>20	>20	>20	>20	>20	>20	NT	>20	>20	>20	>20	>20
	39D	>20	>20	>20	>20	>20	>20	11.1	>20	19.7	17.1	3.27	1.73
4 (a)	45B	>20	>20	>20	>20	>20	>20	>20	>20	>20	>20	3.38	1.51
	50K	>20	>20	>20	>20	>20	>20	>20	>20	>20	>20	>20	>20
	61A	>20	>20	>20	>20	>20	>20	>20	>20	>20	>20	>20	>20
	61B	>20	>20	>20	>20	>20	>20	>20	>20	>20	>20	>20	>20
	23B	>20	>20	>20	>20	>20	>20	NT	>20	>20	>20	>20	>20
	23C	>20	>20	>20	>20	>20	>20	NT	>20	>20	>20	>20	>20
	28A	>20	>20	>20	>20	>20	>20	NT	>20	>20	>20	>20	>20
4 (b) Branch 1	28B	>20	>20	>20	>20	>20	>20	NT	18.2	>20	>20	>20	19.7
	28C	>20	>20	>20	>20	>20	>20	NT	>20	>20	>20	>20	>20
	50F	>20	>20	>20	>20	>20	>20	>20	>20	>20	>20	>20	9.94
	55B	>20	>20	>20	>20	>20	>20	>20	>20	>20	>20	>20	>20
	55C	>20	>20	>20	>20	>20	>20	>20	>20	>20	>20	>20	>20
	39A	>20	>20	>20	>20	>20	>20	>20	>20	>20	>20	>20	>20
	39B	>20	>20	>20	>20	>20	>20	>20	>20	>20	>20	>20	>20
	39C	>20	>20	>20	>20	>20	>20	>20	>20	>20	>20	>20	>20
	45A	>20	>20	>20	>20	>20	>20	>20	>20	>20	>20	>20	>20
4 (b) Branch 2	50B	>20	>20	6.19	13.2	14.8	14.3	3.92	11.2	4.87	>20	>20	8.75
	50C	>20	>20	>20	>20	>20	>20	>20	>20	>20	10.2	2.19	0.3
	50D	>20	>20	>20	>20	>20	>20	>20	>20	>20	>20	>20	>20
	50H	>20	>20	>20	>20	>20	>20	>20	>20	>20	>20	>20	>20
	50J	>20	>20	>20	>20	>20	>20	>20	>20	>20	>20	>20	>20
	55A	>20	>20	>20	>20	>20	>20	>20	>20	>20	>20	>20	>20
	UCA	>20	>20	>20	>20	>20	>20	>20	>20	>20	>20	>20	>20
None	12A-10X	>20	>20	>20	>20	>20	>20	>20	>20	>20	>20	>20	>20
	9A-10X	>20	>20	>20	>20	>20	>20	>20	>20	>20	>20	>20	>20

c

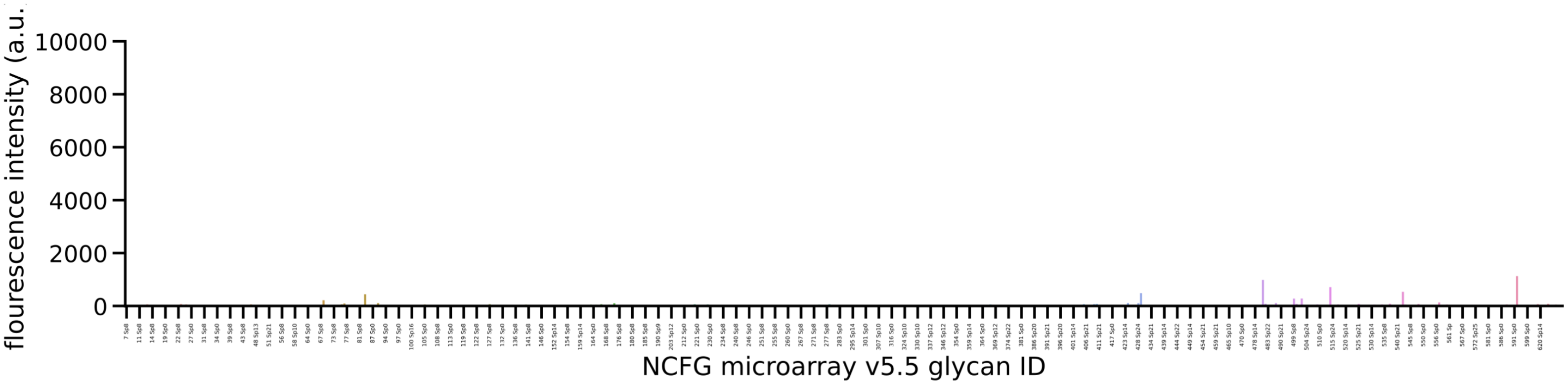

Supplement: S8 Fig — (a) Binding ELISA curves generated with the indicated antibodies and autoantigens. (b) PC39-1 Ab binding EC50 values (μg/mL) to the indicated autoantigens. NT: not tested. (c) Glycan array binding by PC39-1 UCA mAb shows no specific reactivity to glycans. NCFGv5.5: National Center for Functional Glycomics Version 5.5. (PDF) [file ppat.1011416.s008.pdf]
